# Structure–Activity Relationships and Design of Focused Libraries Tailored for *Staphylococcus Aureus* Inhibition

**DOI:** 10.1002/minf.70015

**Published:** 2025-12-10

**Authors:** Alberto Marbán‐González, José L. Medina‐Franco

**Affiliations:** ^1^ DIFACQUIM Research Group Department of Pharmacy School of Chemistry, Universidad Nacional Autónoma de México Mexico City Mexico

**Keywords:** activity cliffs, antibacterials, chemical space, fatty acid synthesis‐II, machine learning, transformation rules

## Abstract

*Staphylococcus aureus* is a bacterium classified among the ESKAPE pathogens, which are anticipated to pose a significant global health emergency in the coming decades. The FabI enzyme, present in both Gram‐positive and Gram‐negative bacteria, is a key enzyme involved in fatty acid synthesis II (FAS‐II). In this study, we utilized transformation rules to expand the chemical space from the most potent *S. aureus* FabI inhibitors. Three newly generated focused libraries, named INDDS, DIADS, and PYRDS, encompassed 172,026 compounds. These compounds were ranked based on structural similarity and predicted pIC_50_ values obtained from machine learning models. This approach allowed to prioritize compounds in each focused library targeting *S. aureus* FabI. We analyzed the pharmacological properties and chemical space diversity of the *S. aureus* FabI inhibitors to gather relevant insights and support the prioritization of compounds for further study. The three newly generated libraries are freely available at https://github.com/DIFACQUIM/S.aureus_inhibitors.

## Graphical abstract

We explored the chemical space of *Staphylococcus aureus* inhibitors and designed focused libraries using transformation rules. Machine learning models were employed to predict pIC_50_ values, and structural similarity to **2**–**4** inhibitors was calculated to rank potential compounds.

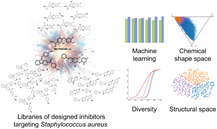

## 1 Introduction

Multidrug‐resistant bacteria are an urgent global concern, affecting both developed countries with thousands of annual deaths and billions in healthcare costs, and it is also a leading cause of death in developing countries. This issue arises due to the inefficient diagnosis of infections and the misuse of broad‐spectrum antibiotics [1]. Some of the pathogens that require urgent research for the development of new antimicrobial agents include the ESKAPE pathogens—*E. faecium*, *S. aureus*, *K. pneumoniae*, *A. baumannii*, *P. aeruginosa*, and *Enterobacter* species. These bacteria are known for their ability to evade the effects of antibiotics and significantly contribute to healthcare‐associated infections. For example, *S. aureus* has developed mechanisms of resistance against inhibitors that interact with penicillin‐binding proteins through horizontal gene transfer, leading to modifications of cell wall proteins [2]. Their rapid acquisition of resistance mechanisms poses a major challenge to global public health. Addressing infections caused by these pathogens is a top priority for developing new antimicrobial therapies. Particularly, methicillin‐resistant *S. aureus* (MRSA) is a significant cause of nosocomial and community‐acquired infections, showing resistance to various antibiotics, including vancomycin, linezolid, and daptomycin [3, 4, 5, 6].

The fatty acid biosynthesis (FAS‐II) pathway has been investigated extensively over the years, and new compounds have been evaluated in clinical trials [7]. Over the past decade, significant efforts have focused on synthesizing and SAR analysis of compounds targeting the FabI enzyme in *S. aureus*. FAS‐II pathway is mediated by several Fab enzymes and is exclusively expressed in bacteria, whereas the FAS‐I pathway is present in humans. Generally, FAS‐II includes transacylases, reductases, isomerases/dehydratases, and synthases, all of which function to elongate fatty acids—integral components of the bacterial cell wall. The most widely distributed enoyl‐ACP reductase (ENR) in bacteria is the FabI enzyme, which catalyzes the reduction of the enoyl‐[acyl‐carrier‐protein]‐(enoyl‐ACP) double bond to produce acyl‐ACP. Disruption of this enzyme's function has been shown to be critical for bacterial survival. Nevertheless, it is necessary to point out some discoveries of the last decade about FabI, specifically in *S. aureus*, an opportunistic pathogen known for its ability to develop multiple resistance mechanisms, including the emergence of FAS‐II bypass mutants. These mutants can incorporate external fatty acids in vitro assays to overcome FAS‐II inhibition by compounds like triclosan, a mechanism also observed in some *Lactobacillales* species. This defense mechanism has been attributed to environmental factors, such as the presence of fatty acids in human fluids, potentially due to hygiene products [8, 9, 10].

**FIGURE 1:**
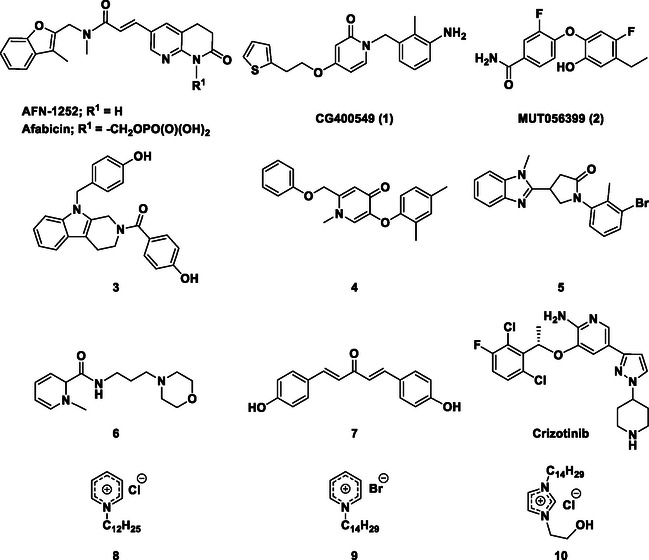
Chemical structures of the most significant or representative inhibitors used against *S. aureus*.

There is substantial research on FabI inhibitors, encompassing diverse chemical scaffolds such as acrylamides, pyridones, diphenyl ethers, tetrahydropyridoindole, pyrrolidine, and others (Figure 1). The prodrug Afabicin is effective against methicillin‐sensitive *S. aureus* (MSSA) and MRSA and currently is developing a phase II clinical trial in the treatment of staphylococcus infections in bone or joint (NCT03723551) (**Debiopharm International SA, 2020**) [11]. CG400549 (**1**) completed Phase II clinical trials to assess its safety in multiple doses and pharmacokinetics in patients with cutaneous abscesses caused by MRSA (NCT01593761) (**CrystalGenomics Inc., 2012**) [12]. In contrast, the compound MUT056399 (**2**) has demonstrated antibacterial activity against Gram‐negative bacteria, including efficacy against methicillin‐ and linezolid‐resistant strains [13]. Unlike Afabicin and **1**, which are specific to staphylococcal infections, **2** has shown broader efficacy in vivo assays and Phase I clinical trials has been completed to evaluate safety and pharmacokinetics (NCT02295813) **(Fab’entech, 2014)** [7, 14, 15, 16]. Some of the most potent inhibitors of FabI discovered to date are based on indole, diphenyl ether, and pyridone cores. For example, the indole‐based inhibitor (**3**, Figure 1) exhibited IC_50_ of 0.1 μM against *S. aureus* FabI and IC_50_ of 4.2 μM against *E. coli* FabI. Additionally, these inhibitors showed antibacterial activity with a minimum inhibitory concentration (MIC) of 0.5 μg/mL for *S. aureus* and 8 μg/mL for *M. catarrhalis* [17]. Inhibitors containing the pyridone structural moiety (**4**) demonstrated an IC_50_ of 0.08 μM, along with MIC values of 0.049 μg/mL for *S. aureus* and MRSA. Furthermore, this inhibitor exhibited a favorable pharmacokinetic profile, supporting their potential for oral administration [18].

In 2024, a comprehensive study was published on the discovery of new inhibitors targeting *S. aureus* FabI as potential treatments for MRSA infections. The racemic mixture **5** in Figure 1 was identified through high‐throughput screening, demonstrating a favorable safety profile in murine models of MRSA‐induced skin infections. Subsequent chiral resolution of **5** revealed that the (*S*)‐enantiomer displayed potent antimicrobial activity, with MIC values ranging from 0.25 to 1 μg/mL and an IC_50_ for FabI of 0.094 μM, whereas the (*R*)‐enantiomer was inactive against MRSA strains. Notably, the (*S*)‐enantiomer consistently reduced the size of skin lesions in infected mice [19].

Computational methods play an important role in identifying bioactive compounds against FabI. A ligand‐based virtual screening (LBVS) was conducted based on the structural similarity of FabI inhibitors. Using 3D similarity models through active conformation alignment, models were selected according to their performance in distinguishing active compounds, achieving a true‐positive rate exceeding 50%. Subsequently, an electrostatic similarity analysis was applied as a second LBVS approach, resulting in two bioactive compounds (**6–** **7**, Figure 1), with inhibition of *S. aureus* exceeding 70% and exhibited MIC values ranging from 125 to 250 μM [20].

In a separate study, a quantitative structure–activity relationship (QSAR) model was constructed from a dataset of 212 compounds. A consensus model was employed to identify compounds with the highest predicted activity in a database of drug‐like ionic liquids generated from synthetic building blocks and reactions. Then, imidazolium and pyridinium compounds were tested against resistant strains of *S. aureus*, including **8**, **9**, and **10**, obtaining inhibition zones with diameters of 19.7–25.3 mm. Nevertheless, determining MIC values can be inconvenient due to multiple protein interactions in bacteria and long chains could be appropriate for InhA enzyme, present in *M. tuberculosis*. It is noteworthy that these ionic compounds contain imidazole and pyridine moieties, which are present in inhibitors of FabI and exhibit activity comparable to the broad‐spectrum antibiotic ceftriaxone [21].

Kuralt and Frlan [22] carried out a cheminformatic analysis on 416 compounds tested with Fab enzymes, where 7% of the active FabI inhibitors were classified as pan‐assay interference compounds [23]. Visualization of the chemical space showed the large diversity of FabI inhibitors, not exclusively targeting *S. aureus*. A matched molecular pair analysis revealed that major structural changes in the chemical structures of the 416 compounds involved hydrophobic groups and uncover 3% of compounds being classified as activity cliffs (ACs)—pairs of highly similar structures with substantial differences in biological activity [24, 25]—indicating a high rate of transformed compounds with similar activity levels. Additional chemoinformatic analysis revealed that features such as Michael acceptors, alkene groups, and tertiary amides increase the probability of activity against FabI [22].

The current limitation of *S. aureus* FabI inhibitors is the emergence of single‐point mutations such as M99T and Y147H, described for **AFN‐1252**. These substitutions confer more than a onefold increase in resistance [26]. Despite the low frequency of resistance, Afabicin's stability in mouse plasma remains a key concern, complicating its evaluation in murine models [27]. MRSA mutants such as A95V display slight resistance to compound **1**, whereas F204S and I193F mutations in MSSA can decrease potency of MIC by two to fourfold, respectively [16].

Given the urgent need for new antistaphylococcal agents due to bacteria's ability to develop resistance mechanisms and evade antibiotics, exploring the diversity and coverage in the chemical space of bioactive compounds is essential. Expanding the chemical space to encompass key interactions in the FabI enzyme that could counteract resistance, together with the availability of rationally designed molecules using systematically transformation rules, may yield structures with improved chemical stability while retaining the structural features of inhibitors that exhibit experimentally validated pharmacodynamic and pharmacokinetic profiles. Herein, we describe the chemical space of *S. aureus* FabI inhibitors that were the basis to generate new chemical structures through transformation rules based on highly active compounds, and the development of libraries focused on FabI. Focused libraries are compound collections designed using ligand‐based insights, where undesirable features are removed, and several structural combinations are systematically explored [28]. As described in this work, we constructed the library by ranking structure similarity and predicted activity using machine learning (ML) models to prioritize the assessment of new antistaphylococcal agents. To the best of our knowledge, there are limited reports of ligand‐based studies that employ similarity models and QSAR using ML in *S. aureus.* To our knowledge, no reports have yet described the use of transformation rules as a strategy to augment the chemical space of FabI inhibitors.

## 2 Methods

**FIGURE 2:**
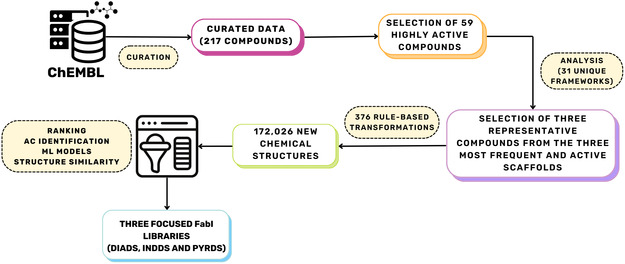
Overview of the methodology of the construction of three focused libraries for *S. aureus* FabI inhibitors. Data was retrieved from ChEMBL via its application programming interface using Python, and subsequently curated to include only valid entries for regression models, to afford a total of 217 compounds. Highly active compounds (above quartile Q_3_ of pIC_50_) were selected to identify unique scaffolds and choose parent structures for application of transformation rules. The generated libraries were curated to remove duplicates to get 172,026 structures. Prediction of pIC_50_ values and similarity scores were then performed on the focused libraries to rank each library.

Figure 2 shows an overview of the methodology to generate focused libraries of inhibitors of *S. aureus* through transformation rules. Overall, compounds with reported activity against FabI were retrieved from ChEMBL 34 [29, 30]. Data curation was done to homogenize biological assay data and fix incorrect structures, yielding 217 compounds. Then, scaffold analysis and literature review of the top 25% of the most potent inhibitors led to the selection of three representative compound inhibitors (**2**, **3**, and **4**). The three selected compounds were subjected to rule‐based transformations, and the newly generated compounds were ranked by predicted values in ML models using different regressors. Structural similarity of 172,026 new transformed compounds with **2**, **3**, and **4** were calculated with a molecular fingerprint, generating three new independent focused libraries tailored to inhibit FabI of *S. aureus*. In the following subsections we provide details of each step.

### 2.1 Dataset Retrieval and Curation

**FIGURE 3:**
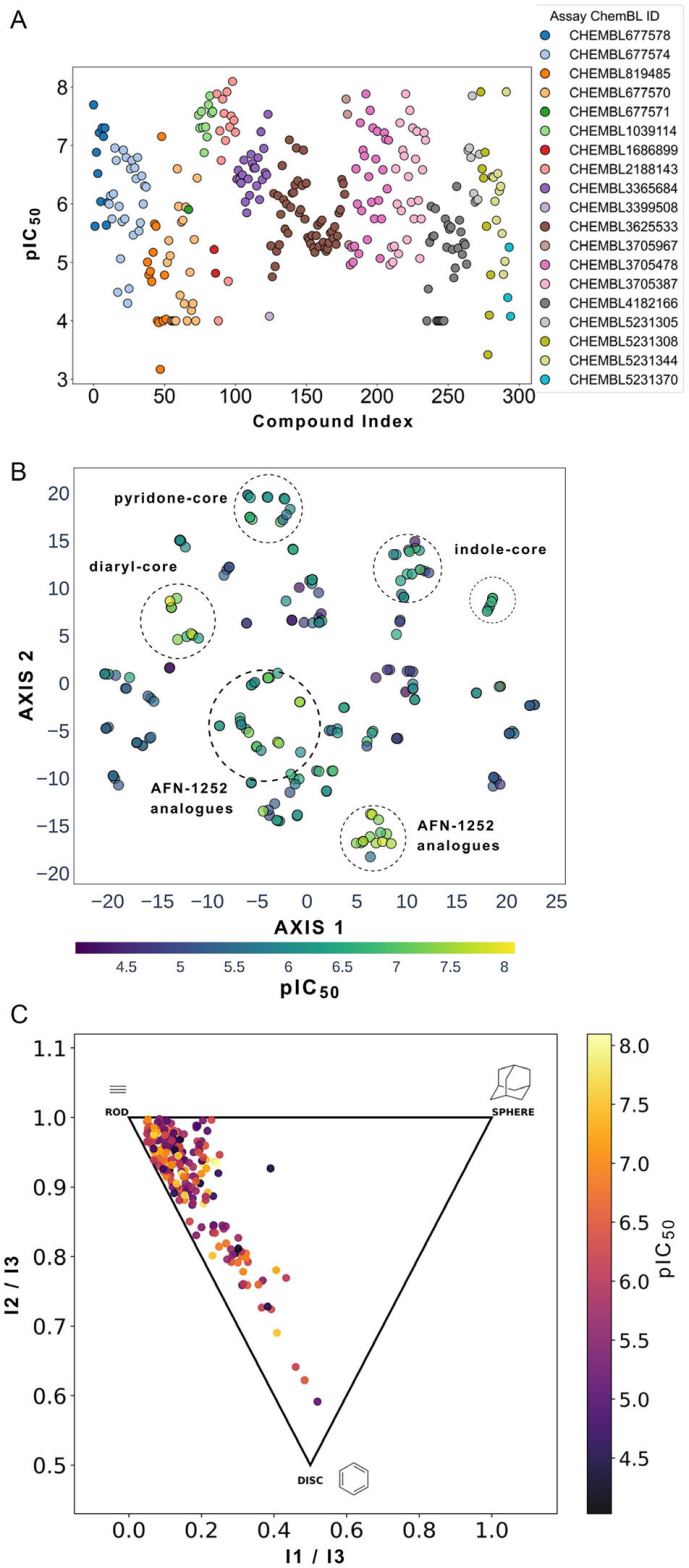
(A) Distribution of *S. aureus* FabI inhibitors across distinct biological assays (Assay ChEMBL IDs), with each color representing a specific assay used to determine the IC_50_ of FabI. The plot shows the most frequently repeated biological assays to measure FabI inhibition. (B) Visualization of the chemical space of 217 *S. aureus* inhibitors using t‐SNE based on MACCS keys (167‐bits) fingerprints and perplexity = 7. The pIC_50_ values are depicted by color: dark blue indicates less active compounds to yellow‐green represents highly active ones. Dashed circles highlight potent diaryl‐, pyridone‐, and indole‐core‐based inhibitors, as well as AFN‐1252 analogs. (C) PMI plot for *S. aureus* inhibitors. Each corner of the triangular area corresponds to a distinct geometry: the top‐left corner represents a rod‐like shape, the top‐right corner indicates a spherical shape, and the bottom corner highlights a disc‐like shape. The pIC_50_ values are depicted by color: black indicates less active compounds; yellow remarks highly active ones.

The biological activities of inhibitors were retrieved from ChEMBL 34 (last updated on 2024‐03‐28) using the application programming interface. The search was performed using the target identifier CHEMBL3994, which corresponds to the FabI enzyme, and was restricted to assays of type “B” to focus specifically on binding assays. Hence, the dataset was filtered to include only inhibitory concentration (IC_50_) data, resulting in a total of 295 entries. The curation process involved analyzing and filtering out duplicate inhibitors (see Supporting Information, Figure S1). In cases where inhibitors were associated with multiple biological assays, the IC_50_ value from the most frequently assay group—depicted in Figure 3A—was selected to ensure consistency (see Supporting Information, Figure S2) and to avoid selecting inhibitors derived from less common assay types. Additionally, a manual review of the original references was conducted to resolve discrepancies in the IC_50_ values of duplicate *S. aureus* compounds recorded in ChEMBL. The unique Simplified Molecular Input Line Entry System (SMILES) strings [31] of the inhibitors were standardized using the open‐source cheminformatics toolkits RDKit [32] implemented in the Spanish cheminformatics GitBook [33], removing salts and metals, correcting and neutralizing charges, and adjusting tautomeric forms. After eliminating erroneous and duplicate entries, molecules with molecular weight (MW) ≥500 Da and pIC_50_ ≤4 yielding 217 inhibitors for *S. aureus* were retained for further analysis.

### 2.2 Dataset Analysis

We analyzed the chemical space of the 217 inhibitors of *S. aureus* curated in Section 2.1. The chemical space was analyzed based on fingerprints and molecular features using t‐SNE and Principal Moments of Inertia (PMI) plots. t‐SNE of two components was performed using the scikit‐learn module [34] to visualize the inhibitors and their potencies [35], using MACCS keys (167‐bits) fingerprints generated with the RDKit package in Python, and a perplexity = 7. A ternary plot of PMI was used to analyze molecular geometry. A low energy conformation was calculated using the MMFF94x force field implemented in Molecular Operating Environment (MOE) 2023, and the three principal moments of inertia, I1, I2 and I3 were determined. Their respective normalized PMI ratios, npr1 = I1/I3 and npr2 = I2/I3, were plotted within a triangular area defined by the vertices (0, 1), (0.5, 0.5) and (1, 1). Each vertex is associated with a molecular shape: rod, disk, and sphere [36].

#### 2.2.1 Scaffold Analysis

We also analyzed the chemical scaffolds of Bemis and Murcko on 217 active compounds against the *S. aureus* dataset (STADS) [37]. The top 25% most active compounds against *S. aureus* FabI were selected from the 217 compounds in the STADS, based on the third quartile (Q_3_) of the pIC_50_ value distribution. This subset consisted of 59 inhibitors, for which their respective Murcko SMILES were determined by the RDKit module to calculate scaffold frequency (Table 1). The most frequently identified framework was (9‐benzyl‐1,3,4,9‐tetrahydro‐2*H*‐pyrido[3,4‐*b*]indol‐2‐yl)(phenyl)methanone (Entry 1, Table 1), followed by diaryl ether (Entry 2, Table 1) and 5‐phenoxy‐2‐(phenoxymethyl)pyridin‐4(1*H*)‐one (Entry 3, Table 1). Notably, these scaffolds represent some of the most potent inhibitors reported in the literature, such as **2**, evaluated in a Phase I clinical trial, and **3** and **4**, with high IC_50_ values of 0.1 μM and 0.08 μM, respectively (Figure 1).

**TABLE 1:** Scaffold analysis from Bemis and Murko approach in the STADS. The mean pIC_50_ and standard deviation of pIC_50_ (SD pIC_50_) were calculated for inhibitors sharing the same scaffold.

Entry	Structure	Amount	Frequency	Mean pIC_50_	SD pIC_50_
1	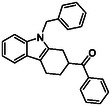	8	13.55	6.77	0.11
2	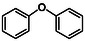	7	11.86	7.44	0.42
3	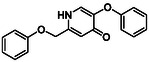	4	6.77	6.90	0.15
4	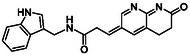	4	6.77	7.27	0.28
5	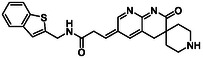	3	5.08	7.57	0.21
6	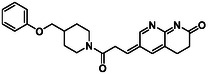	3	5.08	7.14	0.57
7	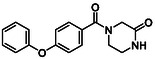	2	3.38	7.67	0.12
8	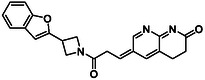	2	3.38	6.77	0.08
9	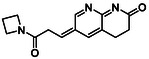	2	3.38	7.18	0.42
10	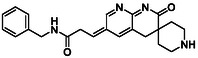	2	3.38	7.36	0.22
11	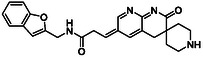	2	3.38	7.50	0.19

### 2.3 Focused Library Design Using Transformation Rules

Construction of the focused library was achieved initially through scaffold analysis, obtaining three representative compounds of the most frequent and active scaffolds determined in section 2.2.1. Herein, the most frequent top three scaffolds were identified lead compounds **2–4** with reported pharmacokinetic and pharmacodynamic profiles in literature, including high IC_50_ and MIC values, adequate solubility, acceptable bioavailability, and suitable parameters for oral administration (volume of distribution, clearance, terminal half‐life, T_max_, and concentration–time curve). As well as minimal inhibition of cytochrome P450 enzymes and adequate toxicological profiles in rats, mice, and dogs, with no concerns regarding maximum tolerated dose, no observed adverse effect level, and no cardiovascular, central nervous system, or respiratory adverse effects [13, 17, 18].

Transformation rules are utilized to expand the chemical space of compounds with shared characteristics, starting from parent structures to generating child structures, under supported modifications. Transformations incorporate bioisosteric replacements, increase metabolic stability or solubility, e.g., by insertion of carboxyl, ester, alcohol, alkyl groups and many others functional groups, as well as conformational restrictions like amide bonds or cyclizations, which are key considerations in drug design. By modifying functional groups while retaining features of the parent structure, transformation rules enable the exploration of new chemical derivatives [38, 39]. In this project, we applied 376 transformation rules to the chemical structures of compounds **2–4**. The transformations consisted of 175 rules initially implemented in MOE 2023, plus 201 additional rules developed by Saldívar‐González et al. [38] to cover transformations involving phenyl rings, heterocycles, amides, anilines, carboxylates, esters, among others, thereby further expanding the chemical space of the selected structures **2–4** and improving physicochemical properties such as membrane permeability, metabolic stability, and bioavailability. The process was carried out in two iterations with no overlap between transformation rules. Examples of these transformations are illustrated in Figure S3 (see Supporting Information), and additional examples can be found in reference [38]. The following restrictions were applied: MW ≤ 500 Da, TPSA between 40 and 140 Å^2^, absence of reactive groups, and synthetic feasibility greater than 0.4 (where 0 indicates unlikely to synthesize and 1 indicates easier to synthesize), implemented in MOE. The application of the transformation rules with the aforementioned restrictions led to a total of 172,026 new chemical structures (Figure 2). Thus, **2** generated structures in a dataset herein named diaryl‐core dataset (DIADS), while **3** and **4** gave rise to the indole‐core dataset (INDDS) and the pyridone‐core dataset (PYRDS), respectively.

#### 2.3.1 Similarity Analysis

To rank the 172,026 molecules generated in Section 2.3, a 2D similarity model was used to compare the chemical structures of the three selected inhibitors of *S. aureus* FabI (**2–4**) with the 172,026 structures derived from the transformation rules. The similarity calculations were done with KNIME Analytics Platform 4.7.0 [40] using the Tanimoto coefficient [41] and the Morgan fingerprint (radius 2, 2048‐bits) [42]. The calculated similarity scores, between **2–4** and DIADS, INDDS and PYRDS datasets, were employed to compare with the predicted pIC_50_ (ppIC_50_) values, calculated with the ML models (described in Section 2.3.3).

#### 2.3.2 Activity Cliffs

Before developing ML models, we analyzed the presence of ACs in STADS with 217 compounds using a Structure‐Activity Similarity (SAS) map. To this end, we computed all 23,436 pair‐wise comparisons using the Tanimoto coefficient and Extended Connectivity Fingerprint of radius 2 (ECFP4) (2048‐bits) (see Supporting Data, Figure S6) [42]. ACs were identified using the Structure–Activity Landscape Index (SALI), defined as follows [43]

$${\text{SALI}}_{i,j}=\frac{\left|A_{i}-A_{j}\right|}{1-\mathrm{sim}\left(i,j\right)}$$

where $A_{i}$ and $A_{j}$ are the activities of the *i*th and *j*th inhibitors, respectively, and sim(*i*,*j*) is the similarity between them. Pairs of compounds with high SALI values are associated with AC. In this work, we analyzed pair‐wise comparisons with structural similarity and activity differences ($\text{Δ}{\mathrm{pIC}}_{50})$ greater than quantile Q_3_ in their distributions, and it was considered as ACs the pair of compounds with SALI values higher than five (see Supporting Data, Table S3).

#### 2.3.3 ML Models

##### 2.3.3.1 Fingerprints

Molecular fingerprints were calculated with MolFeat [44], which includes various fingerprint types such as ECFP [42], Topological torsions [45], Functional Class Fingerprint (FCFP) [42], Atompairs [46], Avalon [47], Layered [48], Pattern [48], and MACCS keys of 167‐bits [49]. The exploration of these fingerprints has been applied in multiple ML models employing diverse drug design approaches, including simpler molecular representations such as Avalon and MACCS keys [50, 51, 52, 53].

##### 2.3.3.2 ML Models

The STADS filtered in section 2.1 was selected to perform ML models to include valid pCI_50_ values and exclude molecules with large MW. Multiple ML models were evaluated using different fingerprints in a nonlinear support vector machine (SVM) regressor (see Supporting Information, Table S4). The data was randomly split into training and testing sets of 75% and 25%, respectively. All fingerprints were standardized to a length of 2048 bits—including ECFP, Topological, FCFP, Atompairs, Pattern, and Layered fingerprints—with the exception of Avalon (512‐bits) and the MACCS keys (167‐bits) fingerprints (see Supporting Information, Figure S7). Further exploration of diverse ML models was conducted on the curated STADS, employing Ridge Linear, Bayesian Ridge Linear, Random Forest (RF), Huber, and K‐Nearest Neighbors (KNN) regressors (see Supporting Information, Figure S8). Hyperparameter optimization was applied to all models (see Supporting Information, Table S5), and their performance was evaluated using the Spearman correlation coefficient for the testing set ($r_{\text{test}}^{2}$) and training set ($r_{\text{training}}^{2}$), as well as mean absolute error (MAE), mean squared error (MSE), and root mean squared error (RMSE). While the correlation coefficients measure the strength of the relationship between predicted and experimental values, the error metrics capture the magnitude of the differences.

##### 2.3.3.3 Validation

Leave‐One‐Out (LOO) cross‐validation was implemented using MSE as the metric to validate model performance. In LOO one compound is removed from the training set, and a new model was generated using the remaining compounds. The process is repeated *n* times, where *n* is the sample size, providing insight into model sensitivity by observing the effect of removing each entry individually. The resulting metric, MSE of cross‐validation (MSE CV), indicates the robustness of the model under this perturbation. Additionally, fivefold CV was applied to the training set to assess the predictive capability of the model. Here, the STADS training set was randomly shuffled and split into five equal‐sized subsets. In each iteration, one subset served as the testing set while the remaining four subsets were used for training [54].

#### 2.3.4 Consensus of Predictive Models to Rank the Focused Libraries

The similarity scores computed in Section 2.3.1 and ML models developed in Section 2.3.3 were used to establish a ranking for the three newly generated datasets: INDDS, DIADS and PYRDS. We calculated ppIC_50_ values for compounds derived from transformation rules using multiple ML regressors (SVM, Ridge, and RF). For each compound, the mean and standard deviation of ppIC_50_ were then calculated across all regressors to establish a consensus metric between SVM, Ridge, and RF models. The mean ppIC_50_ values along with the similarity scores from Section 2.3.1, were then used to rank potential inhibitors based on both predicted biological potency and structural similarity. This process yielded to the three focused libraries targeting *S. aureus* FabI inhibitors: INDDS, DIADS, and PYRDS (Figure 2).

## 3 Results

### 3.1 Dataset Curation

Inhibitors targeting *S. aureus* FabI were retrieved from ChEMBL. Although 59 inhibitors had reported K_i_ values or inhibition percentages, these data types were deemed unsuitable to develop the ML models. K_i_ data were insufficient for building a robust dataset, whereas inhibition percentages reflected bacteria inhibition that add additional variables, decreasing the probability of inhibition against the biological target. Consequently, IC_50_ values for *S. aureus* FabI were used for regression, because these biological assays implied the effect of inhibitors with the target of interest.

During dataset curation, 47 duplicate SMILES were identified (see Supporting Information, Figure S1). To ensure consistency in the STADS dataset, inhibitors were selected from the most frequently used biological assays (e.g., CHEMBL3625533, CHEMBL4182166, CHEMBL3705478, CHEMBL677574, CHEMBL3705387, CHEMBL3365684, CHEMBL677570) as shown in Figure 3A and S2. These assays, which differed in detection methods such as spectrometry or fluorescence (see Supporting Information, Table S1), were prioritized during manual revision of IC_50_ data, enabling the removal of duplicates. Undetermined IC_50_ values of 10 µM were not considered for ML models. 12% compounds exceeding 500 Da and pIC_50_ ≤ 4.0 were also excluded to ensure compliance with Lipinski's and Veber's rules. This curation strategy was essential given the high sensitivity of the data for our predictions with ML models, focusing on drug‐like inhibitors, yielding 217 compounds for STADS.

### 3.2 Dataset Analysis

We employed t‐SNE (Figure 3B and S4) to visualize chemical space and identify compound clusters, while PMI (Figure 3C) was used to evaluate the shape‐likeness of the inhibitors**.** Initially, the most effective way to explore these active compounds involved MACCS keys (167‐bits) and ECFP4 fingerprints (see Supporting Information, Figure S4), using the Multiple Activity Analyzer—MAYA—developed by our research group to visualize the “chemical multiverse” to describe and cluster structural features of *S. aureus* dataset. MAYA is an open‐source tool for compound curation and standardization from SMILES strings with biological activity data. It calculates molecular descriptors (MACCS keys, ECFP4, ECFP6), pharmaceutical‐relevant properties, and pairwise similarity. Dimensionality‐reduction methods such as t‐SNE and Principal Component Analysis (PCA) are used to visualize the “chemical multiverse,” enabling the identification of shared chemical space and clusters based on molecular properties or structural features [55]. Additional methods, such as PCA of STADS showed low variance (data not shown) [56]. In Figure 3B, several noteworthy regions emerged: small clusters of potent diaryl‐, pyridone‐, and indole‐core‐based inhibitors, as well as a larger cluster of AFN‐1252 analogs—all of which exhibited high potency. In the PMI plot shown in Figure 3C, most inhibitors displayed a rod‐like tendency, aligning with the fact that the most potent inhibitors shared this shape, although a few displayed a planar, disc‐like form. This outcome is unsurprising, given that the FabI substrate is an aliphatic chain that remains elongated in the FAS‐II system.

**FIGURE 4:**
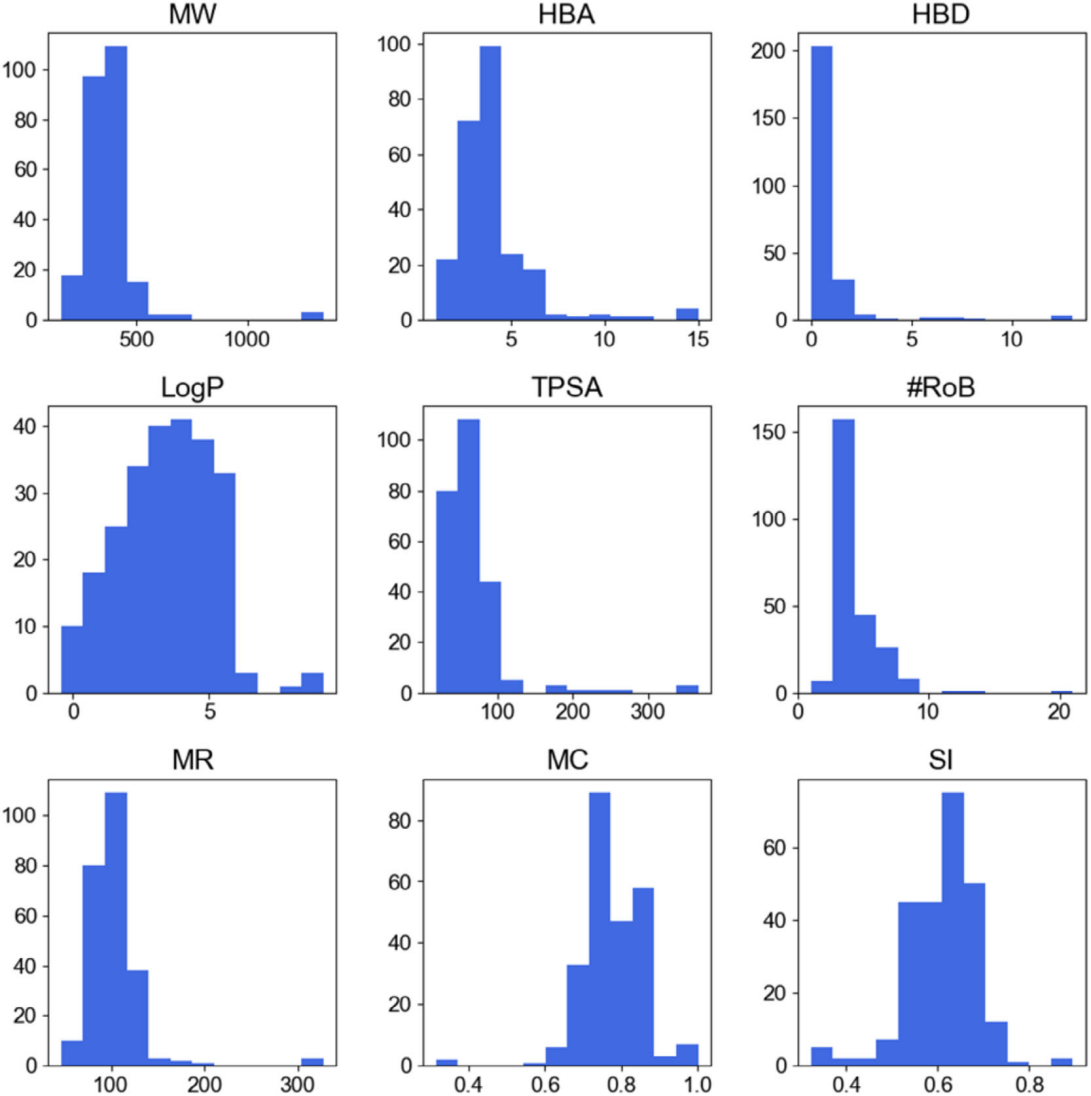
Distribution of descriptors for the *S. aureus* dataset of pharmaceutical interest. Abbreviations: Molecular Weight (MW; g/mol), Hydrogen Bond Donors (HBD), Hydrogen Bond Acceptors (HBA), Topological Polar Surface Area (TPSA; ${Å}^{2}$), Octanol‐Water Partition Coefficient (Log P), Molar Refractivity (MR; ${Å}^{3}/\mathrm{mol}$), Number of Rotatable Bonds (nRoB), Molecular Complexity (MC), and Shape Index (SI).

Figure 4 shows the well‐known empirical rules for drug‐likeness include Lipinski's rule of five (Ro5), which addresses permeability and solubility by constraining MW, hydrogen‐bond acceptors (HBA), hydrogen‐bond donors (HBD), and the octanol–water partition coefficient (LogP) [57]. Veber's rule is associated with drug bioavailability through topological polar surface area (TPSA) and the number of rotatable bonds (#RoB) [58]. Figure 4 provides a full overview of the STADS without filtering by MW and pIC_50_, as described in Section 2.1. In this dataset, most compounds conform to these criteria. Structures that do not comply with drug‐likeness criteria are polyphenol antioxidants, macrocyclic compounds, and fatty acids.

Additionally, relevant descriptors like molecular refractivity (MR) with $\bar{x}=98.28{Å}^{3}/\mathrm{mol}$ correlated with molecular size [59], molecular complexity which is related to the number to stereocenters, fraction of sp^3^ carbon (CSP3 fraction), selectivity and promiscuity [60] had relative high mean value of $\bar{x}=0.76$ compared with $\bar{x}=0.69$ of FDA‐approved drugs retrieved from DrugBank (2025‐01‐14) [61]. Shape index (SI) aligned with the PMI plot, suggesting a balance of branched and linear structural features [62]. Further analysis of the CSP3 fraction revealed a notable presence of aliphatic carbons, frequent heteroatoms and amide bonds (see Supporting Information, Table S2). Moreover, 43.08% of the compounds contained at least one HBD group, an important feature exemplified by the inhibitor **2** (Figure 1) that forms key interactions with Tyr152 in the *S. aureus* FabI active site. Most of these descriptor analyses integrate drug‐likeness criteria for bioavailability and permeability with structural features—ring systems, heteroatoms, polarity, size, and shape—commonly observed among the inhibitors.

#### 3.2.1 Scaffold Analysis

The *S. aureus* FabI dataset has pIC_50_ values with $\bar{x}=6.01$, median $Q_{2}=6.0$, and third quartile $Q_{3}=6.61$ (see Supporting Information, Table S2). The closeness of $\bar{x}\;\text{and}\;Q_{2}$ suggests a relatively symmetric distribution (see Supporting Information, Figure S5). We performed a Bemis & Murcko scaffold analysis on the top 25% most active inhibitors (those above $Q_{3}$), identifying 59 compounds in total and 31 unique frameworks. Because of the dataset size and its structural diversity (visualized in Figure 3B), only the top eleven scaffolds had multiple inhibitors (see Table 1), whereas 20 were unique.

The (9‐benzyl‐1,3,4,9‐tetrahydro‐2*H*‐pyrido[3,4‐*b*]indol‐2‐yl)(phenyl)methanone scaffold (Entry 1, Table 1) appeared most frequently (13.55%) and the skeleton is found in compound **3** (Figure 1). The diaryl ether core (Entry 2, Table 1), exemplified by **MUT056399** (**2**) (Figure 1), followed closely (11.86%), while the 5‐phenoxy‐2‐(phenoxymethyl)pyridin‐4(1*H*)‐one moiety (Entry 3, Table 1) was observed in inhibitor **4** (Figure 1). In addition, several analogs bearing the naphthyridine ring—found in AFN‐1252 or its prodrug Afabicin—contributed 27.07% among the nonunique scaffolds (Entries 4–6, 8, 10–11, Table 1), and many of the unique scaffolds (23.73%) also incorporated this fragment. Hence, the most potent inhibitors primarily involved four scaffolds: (9‐benzyl‐1,3,4,9‐tetrahydro‐2*H*‐pyrido[3,4‐*b*]indol‐2‐yl)(phenyl)methanone, diaryl ether, 5‐phenoxy‐2‐(phenoxymethyl)pyridin‐4(1*H*)‐one, and naphthyridine‐based analogs related to AFN‐1252. Overall, these findings are consistent with the t‐SNE visualization (Figure 3B), where the most active compounds cluster together according to their structure and pIC_50_ values. Most compounds contained more than two rings in STADS—often aromatic, including heterocycles and phenyl moieties—comprising 43.77% saturated and 56.23% unsaturated cycles. This distribution indicates relatively rigid frameworks formed by aromatic rings, aliphatic rings and amide linkages.

### 3.3 Focused Library Design Using Transformation Rules

We selected three representative inhibitors to be the basis to apply the transformation rules. The inhibitor **3** features the (9‐benzyl‐1,3,4,9‐tetrahydro‐2*H*‐pyrido[3,4‐*b*]indol‐2‐yl)(phenyl)methanone scaffold, displaying an *S. aureus* FabI IC_50_ of 379 nM and a MIC of 0.5 µg/mL—properties suitable for antimicrobial development. Moreover, it shows relatively high selectivity for FabI compared with triclosan, which has *S. aureus* FabI IC_50_ of 1100 nM yet a much lower MIC (0.03 µg/mL), likely reflecting triclosan's interaction with multiple targets, thereby reducing its FabI specificity [17].

The second inhibitor selected as a starting point to generate transformation rules was **2** (Figure 1). This compound bearing a diaryl ether structure, demonstrated an *S. aureus* FabI IC_50_ of 12 nM and was active against several *S. aureus* strains (MRSA, MSSA, methicillin‐resistant *S. epidermidis*, and methicillin‐resistant *S. hemolyticus*) with MIC values ranging from 0.03 to 2 µg/mL. Compound **2** also exhibited MIC values below 2 µg/mL against *N. gonorrhoeae*, *N. meningitidis*, *H. influenzae*, *M. catarrhalis*, and *P. mirabilis*, suggesting a potential application as broad‐spectrum target. Additional studies revealed good solubility, strong affinity for human serum albumin, minimal cytochrome P450 inhibition, and low toxicity [13].

The third selected inhibitor, compound **4** contains a 5‐phenoxy‐2‐(phenoxymethyl)pyridin‐4(1*H*)‐one moiety, with *S. aureus* FabI IC_50_ of 80 nM and MIC of 0.049–2 µg/mL for *S. aureus* and MRSA. Pharmacokinetic evaluation showed acceptable values of area under the curve $AUC_{0\to \infty }$, $AUC_{\text{last}}$, terminal half‐life, $C_{\max}$, $T_{\max}$ and estimated bioavailability of 40% [18].

To the three compounds **2–4** we applied transformation rules derived from empirical observations, that allow for the introduction of bioisosteres and fragment substitutions/homologations commonly used to build focused libraries [38]. We considered drug‐likeness filters applying the construction of new molecules below 500 Da, acceptable range of TPSA, no reactive groups, and synthetic feasibility. The structure of inhibitor **3** led to the generation of 85 603 new compounds (referred as INDDS). Similarly, the inhibitor **2** led to the construction of 36 107 new structures (DIADS). Finally, the pyridone‐based inhibitor **4** produced 62 797 new molecules (PYRDS). Each new dataset was curated using the same protocol as for the STADS—including the removal of ionization states introduced by extended transformation rules [38]—and any repeated SMILES were eliminated. Specifically, 6946 duplicates were removed from INDDS, 885 from DIADS, and 4650 from PYRDS, yielding a total of 172 026 structures.

#### 3.3.1 Analysis of Activity Cliffs (ACs)

**FIGURE 5:**
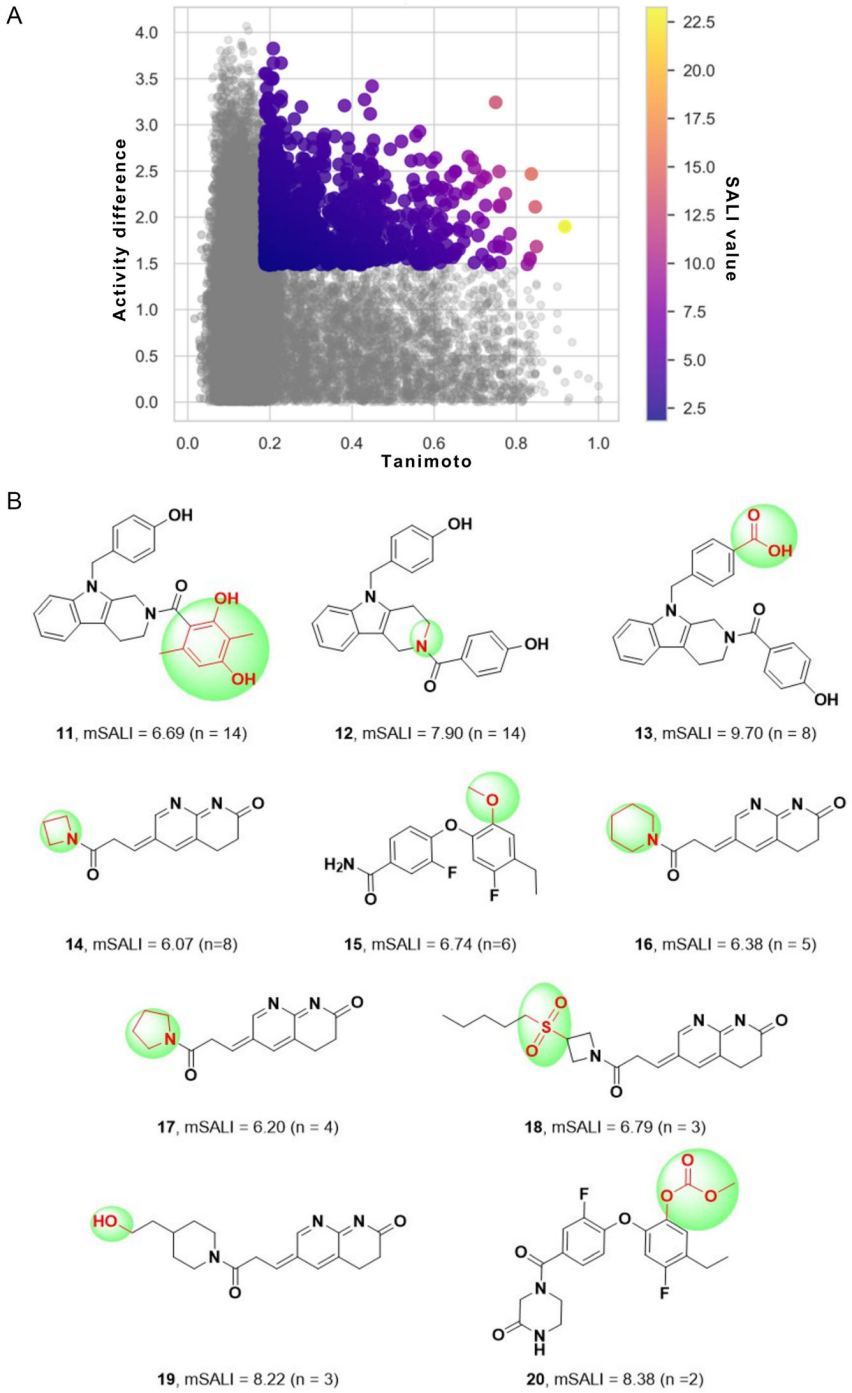
(A) Analysis of SALI values in the SAS map with a 75% threshold corresponding to Q_3_ quantile of activity difference and Tanimoto score, respectively. Each dot represents a pair‐wise comparison of chemical structures in STADS (1137 compound comparisons), plotted using the Tanimoto coefficient (*x*‐axis) calculated with ECFP4 fingerprint and $\text{Δ}{\mathrm{pIC}}_{50}$ (*y*‐axis). SALI values range from 1.8 (purple) to 23.5 (yellow). (B) Identified activity cliffs and their respective mSALI values—mean SALI value comparison of structures in STADS with **11–** **20** structures—, *n* is the frequency of ACs found in the 75% comparison threshold with SALI values higher than five. Key modifications affecting biological activity are highlighted in green regions with atoms and bonds marked in red.

A SAS map plots structure similarity on the *X*‐axis and activity difference ($\text{Δ}{\mathrm{pIC}}_{50})$ on the *Y*‐axis. Each data point represents a pair‐wise comparison. The compounds in STADS were analyzed to visualize ACs in a SAS map. In Figure 5A, data points in the top‐right quadrant represent pairs of compounds with high structural similarity and large potency differences, indicating potential ACs [63]. This quadrant by the structural similarity—calculated with the Tanimoto coefficient and the ECFP4 fingerprint—threshold was set at 0.18, while the $\text{Δ}{\mathrm{pIC}}_{50}$ threshold was 1.46, retaining only compound pairs that exceeded these thresholds and affording 1137 pair‐wise comparisons within the defined region. Structure pairs with SALI values greater than five yielded 78 pair‐wise structures (see Supporting Information, Table S3), overall structural similarity score above 0.5. Figure 5B shows ten compounds classified as ACs based on our criteria. These compounds exhibit different AC classifications: structure **12** is a scaffold cliff; **11**, **13**, **15**, and **20** are R‐group cliffs; and **14**, **16–19** are scaffold/topology cliffs [64]. Notably, compounds **11** and **12** had the most frequent comparisons in this region, with mean SALI (mSALI) values of 6.69 and 7.90, respectively. Compound **11** contains a 2,4‐dihydroxy‐3,6‐dimethylphenyl moiety, which was compared with 14 structurally related compounds featuring diverse substitutions, including 2‐hydroxy‐4‐methylphenyl, 4‐hydroxy‐3‐methylphenyl, 4‐hydroxyphenyl, 4‐methylphenyl, 4‐chlorophenyl, 4‐methoxyphenyl, and phenyl. Similarly, compound **12** shared common comparison structures as **11**. However, compound **11** had a mean $\text{Δ}{\mathrm{pIC}}_{50}=2.27$ and a mean similarity score of 0.65, while compound **12** (scaffold cliff) showed a mean $\text{Δ}{\mathrm{pIC}}_{50}=2.07$ and a mean similarity score of 0.72, highlighting structural similarity features with **3** (similarity score ≈0.83 and $\text{Δ}{\mathrm{pIC}}_{50}=2.46$). These results suggest that isomeric modifications in compound **12** exert a greater impact on biological activity. Additionally, a key modification in compound **13** (R‐group cliff) that shares a similarity score of 0.91 with **3**, where a hydroxyl group is replaced by a carboxylic acid in the 4‐hydroxybenzyl moiety, represents a minor structural change compared to the addition of a tetrasubstituted phenyl ring, as observed in compound **11**. Although the compared structures had diverse modifications, certain pairs—e.g., ACs **15** and **20**—undergo minor changes that significantly affect biological activity, more than two orders of magnitude and similarity scores of ≈0.75. In contrast, compounds **14**, **16**, and **17–19** were compared with inhibitors featuring more extensive modifications in their amide substituents and mostly $\text{Δ}{\mathrm{pIC}}_{50}$ values greater than two (100‐fold biological activity difference) and structure similarities between 0.51 and 0.84.

#### 3.3.2 ML Models

**FIGURE 6:**
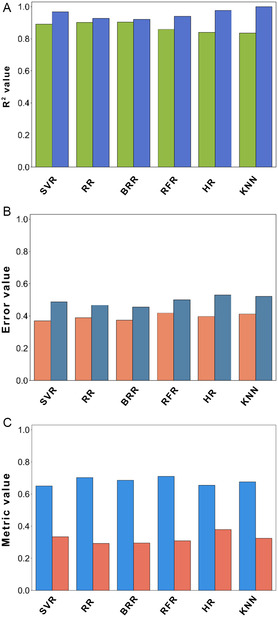
Regression metrics for ML models built from STADS. (A) The yellow‐green bars indicate the Spearman correlation coefficient for the testing set, while the royal‐blue bars represent the Spearman correlation coefficient for the training. (B) The coral bars and steel‐blue bars highlight the MAE and RMSE metrics, respectively. (C) The dodger‐blue and coral bars show the results of CV and LOOCV of the MSE, respectively. ML modes employed were Support Vector Regressor (SVR), Ridge Regressor (RR), Bayesian Ridge Regressor (BRR), Random Forest Regressor (RFR), Huber Regressor (HR), and K‐Nearest Neighbors (KNN).

The initial ML exploration focused on employing descriptors and fingerprints to predict $pIC_{50}$ values for molecules generated through transformation rules. Figure 6A presents the ML models implemented with STADS. Removing identified ACs improved model performance, with the complete removal of ACs reducing overfitting, yielding $r_{\text{test}}^{2}=0.8358$ and $r_{\text{train}}^{2}=0.9759$ on a supporting vector regressor (SVR). Notably, including compounds **11** and **17** further improved the performance, achieving $r_{\text{test}}^{2}=0.8933$ and $r_{\text{train}}^{2}=0.9683$ (see Supporting Information, Figure S7 and Table S4). Across all AC removal experiments, error metrics remained approximately similar.

Figure 6 shows the tested regressors, including Ridge regressor (RR), Bayesian Ridge regressor (BR), RF regressor (RFR), Huber regressor (HR), and KNN, yielded comparable performance, although overfitting was particularly pronounced in HR and KNN models. In contrast, the use of Atompairs, Avalon, Layered, Pattern, and MACCS keys fingerprints declined the model performance and increased overfitting (see Supporting Information, Figures S7 and S8). The FCFP fingerprint performed identically to ECFP4, while topological torsions achieved $r_{\text{test}}^{2}=0.8657$ and $r_{\text{train}}^{2}=0.9407$ (see Supporting Information, Figure S7 and Table S4). These findings suggest that ECFP4, FCFP, or topological torsions are the most suitable fingerprints for building a robust ML model from the current STADS (see Supporting Information, Figure S8). Error metrics such as MAE and MSE, along with validation metrics including LOOCV MSE and CV, remained consistent across all models, as shown in Figure 6B,C see Supporting Information, Figures S7B, S7C, S8B, S8C) . Thus, SVR, RR, and RFR regressors along with ECFP4 fingerprint were used to estimate ${\mathrm{ppIC}}_{50}$ values for the designed libraries.

#### 3.3.3 Focused Libraries

The constructed focused‐libraries INDDS, DIADS, and PYRDS (described in Section 3.3) were systematically analyzed to assess the presence of undesirable structures or properties. For instance, we sought potential ACs among the structures identified from the SAS map, and only ACs **11**, **12**, **13**, **15**, and **20** were used as queries via SMARTS patterns in the RDKit module in Python, as they share a common scaffold with the newly designed libraries (INDDS, DIADS, and PYRDS). In the case of INDDS, no ACs were identified. However, the (1,3,4,9‐tetrahydro‐2*H*‐pyrido[3,4‐*b*]indol‐2‐yl)ethan‐1‐one moiety was retained in ≈66% of the compounds. Rather than discarding the remaining INDDS structures, these compounds can be explored for FabI *S. aureus* inhibition, as alternative modifications to explore SAR—e.g., phosphamide, sulfonamide, tetrazole, triazole, and diazole fragments could be introduced instead of the amide moiety, or modifications of the indole skeleton could be explored, such as 1*H*‐thieno[3,4‐*b*]pyrrole, 1,5‐dihydropyrrolo[3,4‐*b*]pyrrole, 1*H*‐furo[3,4‐*b*]pyrrole, and indolizine, among other structural modifications.

For the DIADS, only 0.88% of the transformed structures can be highlighted as potential ACs, based on compound **15**, revealing that transformation rules serve as a powerful tool for designing and exploring new compounds. Conversely, no ACs were identified in PYRDS; however, 0.42% of the compounds contained the methoxy‐ group as featured by structure **15**. Nevertheless, hydroxyphenyl pyridin‐4(1*H*)‐one moiety is associated with the biological activity as demonstrated by the potent inhibitor **4** described in section 3.3.

Regarding Ro5, the INDDS library showed 0.005% and 6.41% of structures that did not comply with the HBD and LogP criteria, respectively. The DIADS library showed 0.005% and 0.50% noncompliance for HBD and LogP, respectively. The PYRDS library showed 0.26% and 4.20% of structures that did not comply with the HBA and LogP criteria, respectively. Under Veber's rules, the INDDS and DIADS libraries had 0.12% and 0.04% of structures with TPSA > 140 Å^2^, respectively. All PYRDS molecules complied with Veber's rules.

**FIGURE 7:**
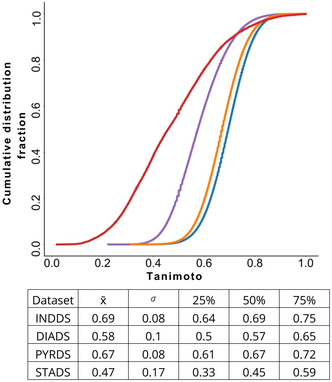
Cumulative distribution function of the pair‐wise similarity values of INDDS, DIADS, PYRDS, and STADS. Similarity was calculated with RDKit employing MACCS keys (167‐bits) and the Tanimoto coefficient. The table summarizes statistics of the calculated similarity scores with the calculated Tanimoto coefficient for the designed compound libraries: 0 indicates highly diverse dataset and 1 indicates less diversity.

Compound diversity, as illustrated in Figure 7, S9 and S10, was assessed by comparing the cumulative distribution functions (CDFs) of INDDS, DIADS, and PYRDS against STADS. The Tanimoto coefficient using MACCS keys (167‐bits), ECFP4 and ECFP6 were employed to construct similarity distributions, enabling quantification and visualization of compound diversity (see Figure 7, S9 and S10). A CDF value closer to zero indicates highly dissimilar structures, while a CDF value near one suggests high structural similarity. Each sample of 5,000 structures from each designed library generated 12,497,500 pair‐wise comparisons. The table in Figure 7 and Table S6 summarizes the CDF statistics, disclosing that INDDS and PYRDS exhibited lower diversity, with Q_2_ values in close proximity. In contrast, DIADS was more structurally dissimilar with Q_2_ = 0.57, whereas STADS displayed the greatest diversity with Q_2_ = 0.45. These diversity patterns align with the expansion of the chemical space from parent structures **2–4**, where child compounds share structural features, resulting in lower diversity relative to the scaffold structures, as highlighted by STADS diversity. Moreover, the greater diversity observed in DIADS compared to INDDS and PYRDS can be attributed to the fact that over one hundred of the applied transformation rules involved phenyl modifications.

**FIGURE 8:**
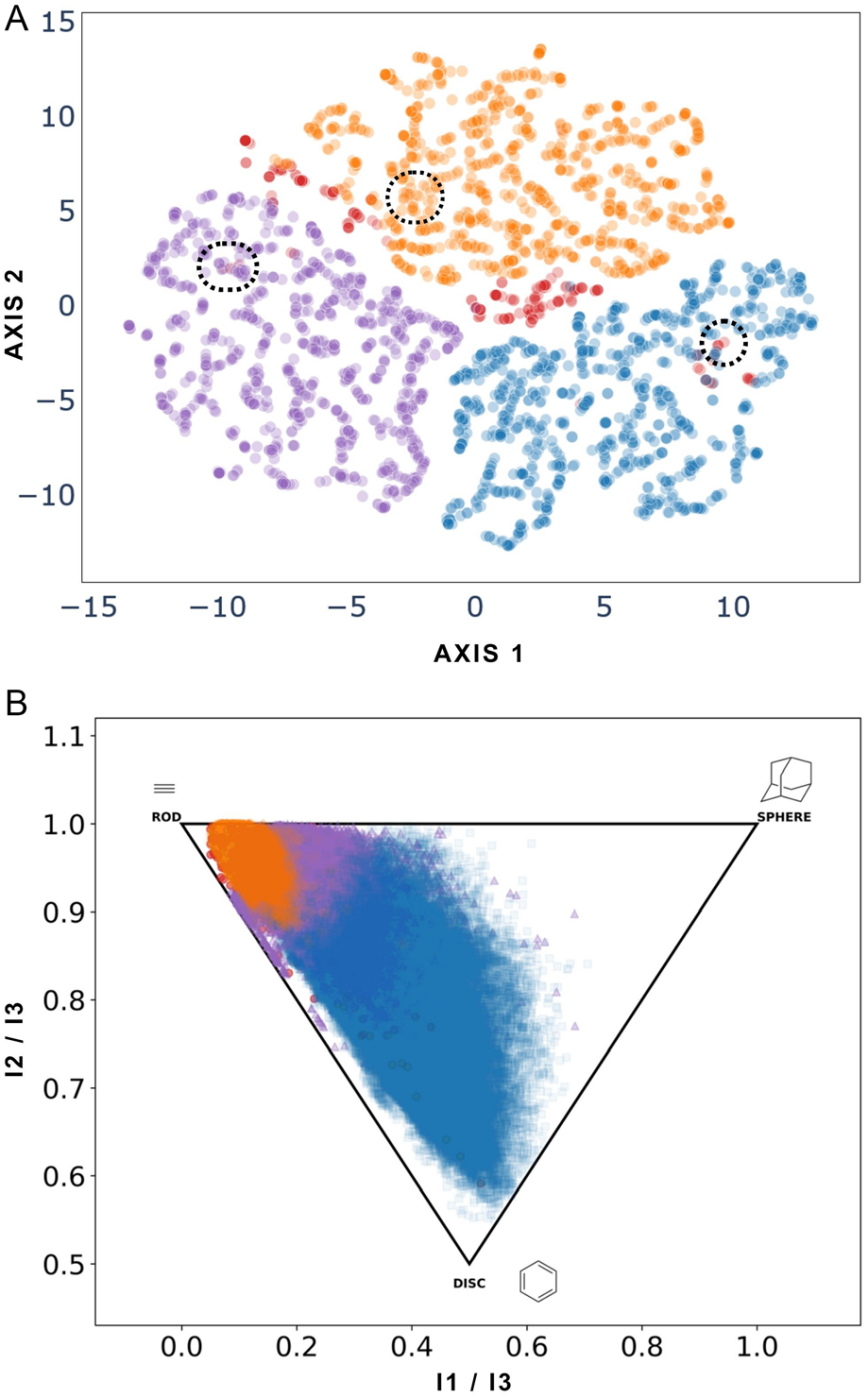
(A) Visualization of the chemical space of designed libraries (INDDS, DIADS, and PYRDS) using t‐SNE based on MACCS keys (167‐bits) fingerprints calculated with RDkit. Dashed circles indicate relative positions of the parent structures **2–4**. (B) PMI plot for designed libraries (INDDS, DIADS, and PYRDS). Each corner of the triangular area corresponds to a distinct geometry: the top‐left corner represents a rod‐like shape, the top‐right corner indicates a spherical shape, and the bottom corner highlights a disc‐like shape. The points were colored by dataset: INDDS (blue), DIADS (purple), PYRDS (orange), and STADS (red).

Figure 8 presents a 2D visual representation of the chemical space based on molecular structures, using Tanimoto coefficient (MACCS keys, 167‐bits) to visualize the structural expansion of 1000 randomly selected compounds from each designed library. The overlap between INDDS, DIADS, and PYRDS with STADS indicates that transformation rules expand the structural space from parent compounds **2–4**. Furthermore, the PMI plot confirmed that the molecular shape of the generated compounds is mostly preserved, exhibiting a preference for rod and disk‐like shapes, consistent with STADS in Figure 3C.

**FIGURE 9:**
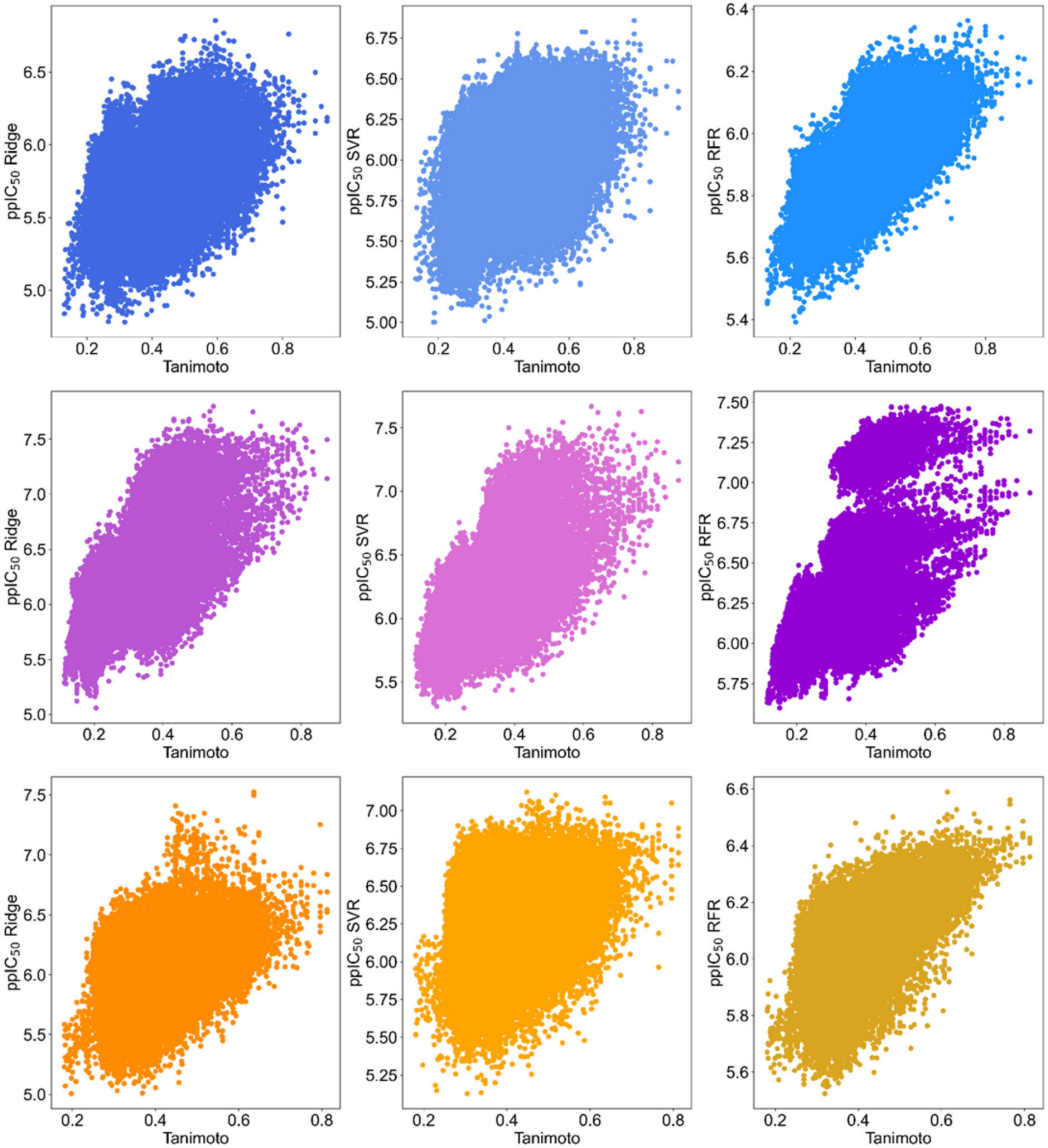
Comparison of ppIC_50_ (*y*‐axis) calculated by RR (first column), SVR (second column), and RFR (third column) against similarity scores (*x*‐axis, using a Morgan fingerprint of 2048‐bits) for each designed library: INDDS (blue colors), DIADS (purple colors) and PYRDS (orange colors). 0 indicates that the designed libraries are less similar to parent structures, while 1 remarks similar compounds to parent structures **2–** **4**, respectively. Greater predicted values of ppIC_50_ mean more potency. The estimated Pearson correlation coefficients were as follows: INDDS (first row): SVR (r^2^ = 0.381), RR (r^2^ = 0.413), RFR (r^2^ = 0.649); DIADS (second row): SVR (r^2^ = 0.691), RR (r^2^ = 0.661), RFR (r^2^ = 0.647); PYRDS (third row): SVR (r^2^ = 0.460), RR (r^2^ = 0.310), RFR (r^2^ = 0.593).

In Figure 9, the structural similarity of each compound in the focused libraries was compared with its parent structures **2–4**, which were used to expand the chemical space via transformation rules. Additionally, ppIC_50_ values obtained using SVR, RR, and RFR were compared to assess the association between ML model predictions (*X*‐axis) and the structure similarity (*Y*‐axis) computed with the Tanimoto coefficient and MACCS keys. The INDDS library (first row in Figure 9), where the Pearson correlation between similarity scores and ppIC_50_ was 0.38 for SVR and 0.41 for RR. A stronger correlation of 0.64 was observed for RFR, indicating a higher relationship between ppIC_50_ predictions and structure similarity. For the DIADS library (second row in Figure 9), correlation coefficients were similar across all regressors, ranging from 0.61 to 0.69. The PYRDS library exhibited (third row in Figure 9) a performance trend comparable to INDDS. The correlation coefficients were 0.31 for SVR and 0.46 for RR, while RFR demonstrated a stronger correlation of 0.59. From the comparisons of structure similarity to the parent compound vs. calculated activity presented in Figure 9 can be deduced that molecules with both high similarity and predicted activity (top‐right quadrant) represent molecules that can be further prioritized for experimental screening. Compounds with high predicted activity but lower structure similarity to the parent compound (top‐left quadrant) represent scaffold‐hops, which can also be prioritized for experimental screening, although they are expected to be structurally distinct from the parent molecules.

**FIGURE 10:**
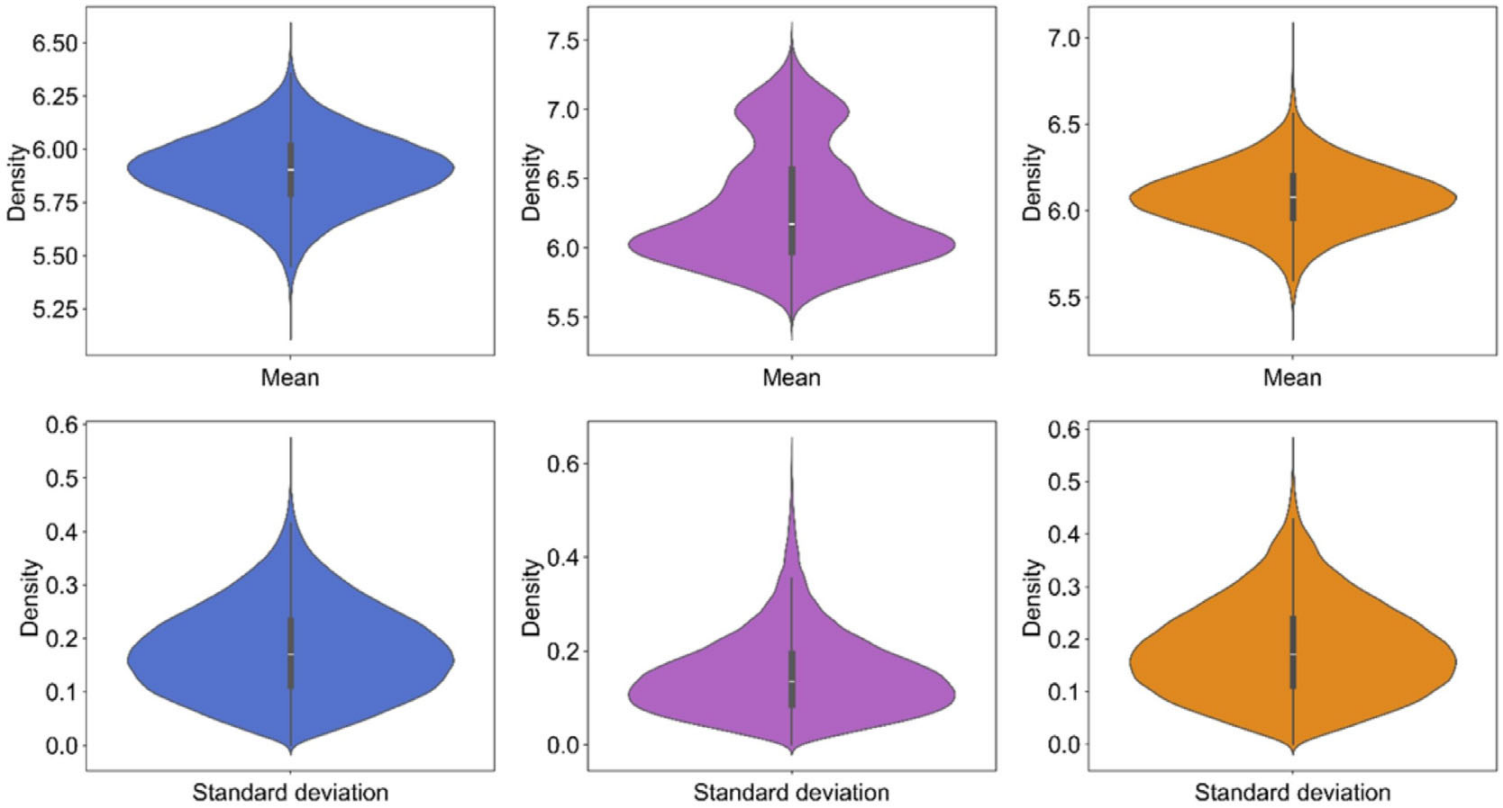
Violin plots of mean values (first row) and standard deviation (second row) of consensus of SVR, RR, and RFR models utilized by the three designed libraries INDDS, DIADS, and PYRDS. Standard deviation highlights the consistency across the models, exhibiting σ values less than 0.55, 0.63 and 0.53 for INDDS, DIADS and PYRDS, respectively. INDDS ($\bar{x}=5.9004,\sigma =0.1719$), DIADS ($\bar{x}=6.2922,\sigma =0.4181$), PYRDS ($\bar{x}=6.0808,\sigma =0.1850$). The distributions were colored by dataset: INDDS (blue), DIADS (purple), PYRDS (orange), and STADS (red).

Figure 10 and S11 presents violin and distribution plots depicting the mean and standard deviation of ppIC_50_ values across the ML models. The mean $\bar{x}$ of all regressors for each library were 5.90, 6.29, and 6.08 for INDDS, DIADS, and PYRDS, respectively. The standard deviation ranged between 0.17 and 0.41. These findings indicate that ppIC_50_ values were consistent across regressors, as their standard deviations remained below 0.63. This consistency motivated us to rank the designed compounds based on their ppIC_50_ values and respective similarity scores to prioritize their exploration in drug discovery. The designed libraries exhibited a low proportion of ACs. Diversity analysis, measured through the CDF, showed lower diversity compared to STADS, confirming that our libraries are focused on structural and molecular shape features to expand the chemical space supported by highly active compounds **2–4**.

## 4 Conclusions

In this study we generated three novel virtual libraries, totaling 176 026 compounds, with the aim to expand the chemical space of the most potent *S. aureus* FabI inhibitors. The library design was based on the application of transformation rules and regressors models. The three newly generated libraries are freely available to the community for further in silico or experimental screening. Of note, this is the first study to implement transformation rules for the design of FabI inhibitors. Chemical space analysis of the STADS dataset enabled the identification of clusters composed of diaryl‐, pyridone‐, and indole‐core‐based compounds, as well as AFN‐1252 analogs. Additionally, PMI analysis revealed a predominant rod‐like molecular geometry. Most compounds in STADS complied with Ro5 and Veber's rules for bioavailability and permeability, exhibiting structural features such as aliphatic carbons, heteroatoms, heterocycles, phenyl moieties, and amide bonds. The scaffold analysis identified 31 distinct scaffolds, with the most representative being (9‐benzyl‐1,3,4,9‐tetrahydro‐2*H*‐pyrido[3,4‐*b*]indol‐2‐yl)(phenyl)methanone), diaryl ether, and 5‐phenoxy‐2‐(phenoxymethyl)pyridin‐4(1*H*)‐one. Representative inhibitors **2**–**4** were selected from these scaffold clusters based on their pharmacokinetic suitability, selectivity, and high potency. **2**–**4** inhibitors were subjected to transformation rules to expand the chemical space. The newly generated compounds were constrained by MW limits, TPSA range, the exclusion of reactive groups, and moderate‐to‐easy synthetic feasibility. This process yielded 85,603, 36,107, and 62,797 compounds for INDDS, DIADS, and PYRDS, respectively. The approach presented in this work generated 172,026 new structures, with basically no overlap (only 0.009%, see Supporting Information, Table S7) with the raw dataset (STADS). Furthermore, potential feature of AC **15** was found in only 0.88% of the DIADS library, while ACs **11**–**14** and **16**–**20** were not present in any of the designed libraries, demonstrating an effective paradigm for expanding chemical space through structural modifications supported by drug design strategies to improve pharmacokinetic and pharmacodynamic properties.

DIADS reflected the greatest diversity due to scaffold phenyl‐modifications, while INDDS and PYRDS displayed lower diversity. The preservation of molecular shape was confirmed by PMI, and the expansion of structural diversity was featured with t‐SNE visualization. SVR, RR, and RFR models exhibited the best performance in reducing overfitting and were employed to calculate ppIC_50_ values for the designed libraries. The combination of ppIC_50_ predictions and structural similarity provided a rational approach to ranking the designed molecules, offering a valuable resource to the scientific community for further computational analysis, their synthesis and biological evaluation of the newly designed libraries.

## Supporting Information

Additional supporting information can be found online in the Supporting Information section. **Supporting** **Fig. S1:** Histogram of repeated SMILES in the dataset of inhibitors against *S. aureus* FabI. **Supporting** **Fig. S2:** Histogram of ChEMBL assay IDs for inhibitors against *S. aureus* FabI in the STADS. **Supporting** **Fig. S3:** Examples of transformation rules applied to structures **2–4**. The parent structure **2** was modified using transformation rules to improve metabolic stability (row 1) or through bioisosteric replacement (row 2), as reported in drug design. Structure **4** was altered to increase polarity (row 3). The final examples show structures generated from parent structures **4** and **3**, where, in a second iteration, the transformations were aimed at reducing hydrophobicity and at performing a bioisosteric replacement, respectively. **Supporting** **Fig. S4:** Visualization of the chemical space of STADS using t‐SNE based on ECFP4 (r = 2, 2048‐bits) fingerprint calculated with RDkit. Dots were colored by pIC_50_. **Supporting** **Fig. S5:** Distribution and dispersion plots of descriptors for the *S. aureus* dataset. Abbreviations: Molecular Weight (MW; g/mol), Hydrogen Bond Donors (HBD), Hydrogen Bond Acceptors (HBA), Topological Polar Surface Area (TPSA; Å^2^), Octanol‐Water Partition Coefficient (Log P), Molar Refractivity (MR; Å^2^/mol), Number of Rotatable Bonds (nRoB), Molecular Complexity (MC), and Shape Index (SI). **Supporting** **Fig. S6:** Analysis of structure similarity in the dataset of inhibitors of *S. aureus* FabI given by Tanimoto coefficient using ECFP4 (r = 2, 2048‐bits). **Supporting** **Fig. S7:** Regression metrics for a support vector regressor built from *S. aureus* dataset, employing different fingerprints. A) The yellow‐green bars indicate the Spearman correlation coefficient for the testing set, while the royal‐blue bars represent the Spearman correlation coefficient for the training set. B) The coral bars and steel‐blue bars highlight the MAE and RMSE metrics, respectively. C) The dodger‐blue and coral bars show the results of cross‐validation and leave‐one‐out cross‐validation of the MSE, respectively. **Supporting** **Fig. S8:** Regression metrics for machine learning models built from *S. aureus* dataset, employing ECFP, Topological torsions, Atompairs and MACCS keys fingerprints. A) The yellow‐green bars indicate the Spearman correlation coefficient for the testing set, while the royal‐blue bars represent the Spearman correlation coefficient for the training set. B) The coral bars and steel‐blue bars highlight the MAE and RMSE metrics, respectively. C) The dodger‐blue and coral bars show the results of cross‐validation and leave‐one‐out cross‐validation of the MSE, respectively. **Supporting** **Fig. S9:** Cumulative distribution function of the pair‐wise similarity values of INDDS, DIADS, PYRDS and STADS. Similarity was calculated with RDkit employing ECFP4 (r = 2, 2048‐bits) and the Tanimoto coefficient. **Supporting** **Fig. S10:** Cumulative distribution function of the pair‐wise similarity values of INDDS, DIADS, PYRDS and STADS. Similarity was calculated with RDkit employing ECFP6 (r = 3, 2048‐bits) and the Tanimoto coefficient. **Supporting** **Fig. S11:** Distributions of mean $\bar{x}$ values (first row) and standard deviation (second row) of SVR, RR and RFR models utilized by the three designed libraries INDDS, DIADS, and PYRDS. INDDS, DIADS, PYRDS. INDDS, DIADS, and PYRDS. INDDS ($\bar{x}$ = 5.9004, σ = 0.1719), DIADS ($\bar{x}$ = 6.2922, σ = 0.4181), PYRDS ($\bar{x}$ = 6.0808, σ = 0.1850). **Supporting** **Table S1:** Description of assays used to measure the IC_50_ of *S. aureus* FabI by Assay ChEMBL ID. **Supporting** **Table S2:** Statistics of significant pharmaceutical properties and constitutional descriptors in STADS. **Supporting Table S3:** Pair‐wise comparison of chemical structures in STADS with the highest similarity (Tanimoto coefficient) and SALI values greater than five, using ECFP4 fingerprint and, ΔpIC_50_ as utilized in the SAS MAP with a threshold of 75% (quantiles Q_3_ of ECFP4 and ΔpIC_50_). **Supporting** **Table S4:** Regression metrics for a support vector regressor built from STADS using different fingerprints. **Supporting** **Table S5:** Hyperparameter optimization in the ML models. R^2^ metrics and CV validation were assessed using the Spearman correlation coefficient. **Supporting Table S6:** Summary statistics of the similarity scores calculated using the Tanimoto coefficient for the designed compound libraries and STADS from cumulative distribution functions. **Supporting Table S7:** Generated structures obtained from transformation rules and their presence in the original STADS with ACs. Experimental pIC_50_ and mean ppIC_50_ of all ML models (SVR, RR and RFR) are described along with similarity scores calculated from parent structures **2–4**.

## Funding

This work was supported by DGAPA, UNAM, Programa de Apoyo a Proyectos de Investigación e Innovación Tecnológica (PAPIIT), (IG200124).

## Conflicts of Interest

The authors declare no competing financial interest.

## Supporting information

Supplementary Material

## Data Availability

The project code and the libraries are available at https://github.com/DIFACQUIM/S.aureus_inhibitors. All the necessary packages were integrated to be installed in Jupyter notebook. Input files and datasets of the designed libraries can be found on the GitHub repository.
