# Supplementary material for: Structure–Activity Relationships and Design of Focused Libraries Tailored for Staphylococcus Aureus Inhibition
Source: Mol Inform. 2025 Dec 10;44(11-12):e70015. doi: 10.1002/minf.70015 (PMC12694758; doi:10.1002/minf.70015)
Supplement: Supplementary file 1 — Supplementary Material [file MINF-44-e70015-s001.pdf]

Supporting Information

Structure-activity Relationships and Design of Focused Libraries Tailored for *Staphylococcus aureus* Inhibition

Alberto Marbán-González, José L. Medina-Franco

DIFACQUIM Research Group, Department of Pharmacy, School of Chemistry, Universidad Nacional Autónoma de México, Avenida Universidad 3000, Mexico City 04510, Mexico

Contents

		Page
**Figure S1**	Histogram of repeated SMILES in the dataset of inhibitors against *S. aureus* FabI.	S3
**Figure S2**	Histogram of ChEMBL assay IDs for inhibitors against *S. aureus* FabI in the STADS.	S3
**Table S1**	Description of assays used to measure the IC_{50} of *S. aureus* FabI by Assay ChEMBL ID.	S4
**Figure S3**	Examples of transformation rules employed on structures 2-4.	S6
**Figure S4**	Visualization of the chemical space of STADS using t-SNE based on ECFP4 fingerprint calculated with RDkit. Dots were colored by pIC_{50}.	S7
**Table S2**	Statistics of significant pharmaceutical properties and constitutional descriptors in STADS.	S8
**Figure S5**	Distribution and dispersion plots of descriptors for the *S. aureus* dataset.	S9
**Figure S6**	Analysis of structure similarity in the dataset of inhibitors of *S. aureus* FabI given by Tanimoto coefficient using ECFP4 (r = 2, 2048-bits).	S10
**Table S3**	Pair-wise comparison of chemical structures in STADS with the highest similarity (Tanimoto coefficient) and SALI values greater than five, using ECFP4 fingerprint and ΔpIC_{50}, as utilized in the SAS MAP with a threshold of 75%.	S11
**Table S4**	Regression metrics for a support vector regressor built from STADS using different fingerprints.	S14
**Figure S7**	Regression metrics for a support vector regressor built from *S. aureus* dataset, employing different fingerprints.	S15
**Figure S8**	Regression metrics for machine learning models built from *S. aureus* dataset, employing ECFP, Topological torsions, Atompairs and MACCS keys fingerprints.	S16
**Table S5**	Hyperparameter optimization in the ML models. R^2 metrics and CV validation were assessed using the Spearman correlation coefficient.	S17

**Figure S9**	Cumulative distribution function of the pair-wise similarity values of INDDS, DIADS, PYRDS and STADS. Similarity was calculated with RDkit employing ECFP4 and the Tanimoto coefficient.	S18
**Figure S10**	Cumulative distribution function of the pair-wise similarity values of INDDS, DIADS, PYRDS and STADS. Similarity was calculated with RDkit employing ECFP6 and the Tanimoto coefficient	S18
**Table S6**	Summary statistics of the similarity scores calculated using the Tanimoto coefficient for the designed compound libraries and STADS from cumulative distribution functions.	S19
**Table S7**	Generated structures obtained from transformation rules and their presence in the original STADS with ACs. Experimental pIC_{50} and mean $ppIC_{50}$ of all ML models (SVR, RR and RFR) are described along with similarity score calculated from parent structures **2-4.**	S20
**Figure S11**	Histograms of mean values (first row) and standard deviation (second row) of SVR, RR and RFR models utilized by the three designed libraries INDDS, DIADS, and PYRDS.	S22

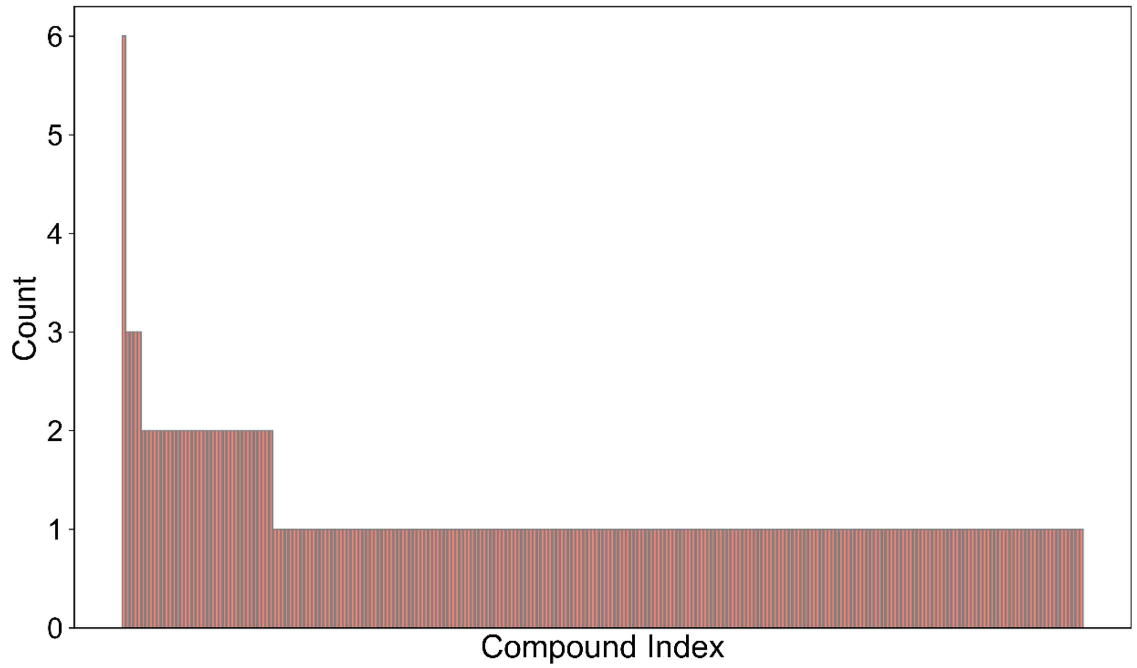

Figure S1. Histogram of repeated SMILES in the dataset of inhibitors against *S. aureus* FabI.

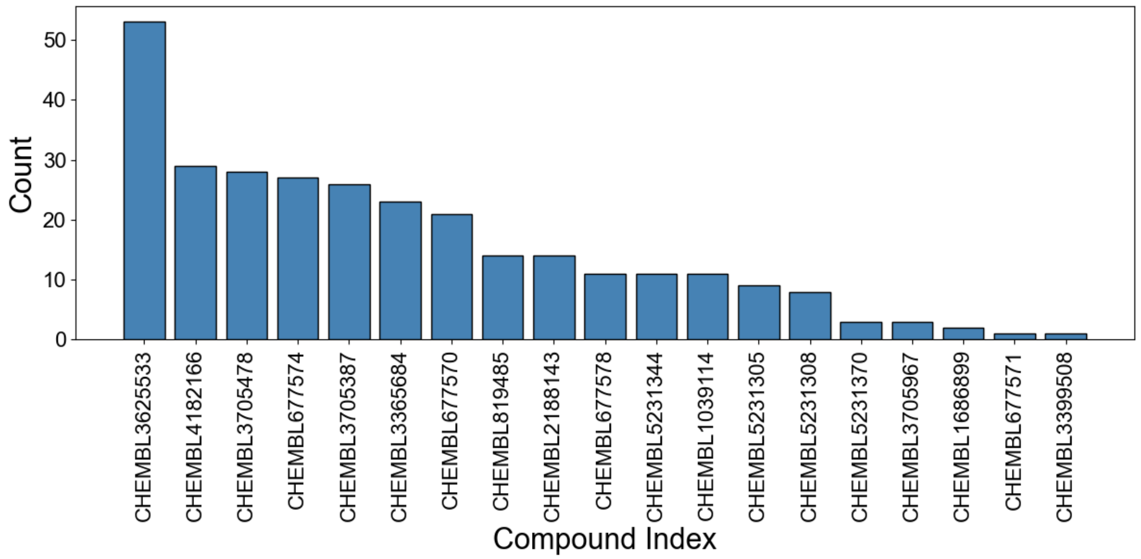

Figure S2. Histogram of ChEMBL assay IDs for inhibitors against *S. aureus* FabI in the STADS.

Table S1. Description of assays used to measure the IC_{50} of *S. aureus* FabI by Assay ChEMBL ID.

Assay description	Assay ChEMBL ID
Inhibitory activity against Enoyl-ACP reductase FabI in Staphylococcus aureus	CHEMBL677578
Antibacterial activity against Staphylococcus aureus FabI	CHEMBL677574
Concentration required for the 50% inhibition of enoyl-ACP reductase from Staphylococcus aureus was determined.	CHEMBL819485
Evaluated for inhibition of enoyl acyl carrier protein reductase (FabI) in Staphylococcus aureus.	CHEMBL677570
Evaluated for inhibition of enoyl acyl carrier protein reductase (FabI) of Staphylococcus aureus in the presence of NADH using crotonoyl CoA as substrate	CHEMBL677571
Inhibition of Staphylococcus aureus FabI	CHEMBL1039114
Inhibition of Staphylococcus aureus enoyl-ACP reductase assessed as increase of NADPH level	CHEMBL1686899
Inhibition of Staphylococcus aureus recombinant FabI using trans-2-octenoyl N-acetylcysteamine thioester as substrate preincubated for 60 mins	CHEMBL2188143
Inhibition of Staphylococcus aureus FabI-mediated reduction of enoyl-ACP preincubated for 30 mins measured after 2 hrs by spectrophotometry	CHEMBL3365684
Inhibition of Staphylococcus aureus FabI-mediated trans-2-octenoyl N-acetylcysteamine (t-o-NAC thioester) substrate reduction assessed as decrease in NADPH by ELISA	CHEMBL3399508
Inhibition of Staphylococcus aureus FabI assessed as reduction in inhibition of reduction of trans-2-octenoyl N-acetylcysteamine substrate by spectrophotometry	CHEMBL3625533
Inhibition Assay: The assay buffer AB contained 50 mM ADA (N-(2-acetamido)iminodiacetic acid monosodium salt) pH 6.5, 1 mM dithiothreitol, 0.006% Triton-X100 and 50 mM NaCl. The following components are added in a white polystyrene Costar plate (Ref 3912) up to a final volume of 55.5 uL: 1.5 uL DMSO or inhibitor dissolved in DMSO and 54 uL of a FabI/NADPH/NADP+ mixture in AB. After 60 min of pre-incubation at room temperature, the reaction is started by addition of 5 uL of trans-2-octenoyl N-acetylcysteamine thioester (t-o-NAC) to a final volume of 60.5 uL. This reaction mixture is then composed of 2 nM FabI, 40 uM NADPH (Sigma, N7505), 10 uM NADP+ (Sigma, N5755), 100 uM t-O-NAC and compound at defined concentration. Fluorescence intensity of NADPH (lamda=ex=360 nm, lamda=em=520 nm) is measured immediately after t-O-NAC addition (T0), and approximately 50 min later (T50) by a Fluostar Optima (BMG).	CHEMBL3705967
Fluorescence Based Assay: Compound inhibitory activity of FabI enzyme is measured in vitro by the IC5 so determination using a fluorescence based assay.The protein FabI from S. aureus is prepared and purified using standard methods for recombinant protein expression after cloning of the gene in a prokaryotic expression vector.The biochemical activity of the FabI enzyme is assessed using the following method.The assay buffer AB contained 50 mM ADA (N-(2-acetamido)iminodiacetic acid monosodium salt) pH 6.5, 1 mM dithiothreitol, 0.006% Triton-X100 and 50 mM NaCl.The following components are added in a white polystyrene Costar plate (Ref 3912) up to a final volume of 55.5 uL: 1.5 uL DMSO or inhibitor dissolved in DMSO and 54 uL of a FabI/NADPH/NADP+ mixture in AB. After 60 min of pre-incubation at room temperature, the reaction is started by addition of 5 uL of trans-2-octenoyl N-acetylcysteamine thioester (t-o-NAC) to a final volume of 60.5 uL.	CHEMBL3705478
Fluorescence Based Assay: The assay buffer "AB" contained 50 mM ADA (N-(2-acetamido)iminodiacetic acid monosodium salt) pH 6.5, 1 mM dithiothreitol, 0.006% Triton-X100 and 50 mM NaCl. The following components are added in a white polystyrene Costar plate (Ref 3912) up to a final volume of 55.5 uL: 1.5 uL DMSO or inhibitor dissolved in DMSO and 54 uL of a FabI/NADPH/NADP+ mixture in AB. After 60 min of pre-incubation at room temperature, the reaction is started by addition of 5 uL of trans-2-octenoyl N-acetylcysteamine thioester (t-o-NAC) to a final volume of 60.5 uL. This reaction mixture is then composed of 2 nM FabI, 40 uM NADPH (Sigma, N7505), 10 uM NADP+(Sigma, N5755), 100 uM t-O-NAC and compound at defined concentration. Fluorescence intensity of NADPH (lamda=ex=360 nm, lamda=em=520 nm) is measured immediately after t-O-NAC	CHEMBL3705387

addition (T0), and approximately 50 min later (T50) by a Fluostar Optima (BMG) so as to achieve ~30% of NADPH conversion.	
Inhibition of Staphylococcus aureus subsp. aureus Rosenbach ATCC 43300 FabI using crotonyl-CoA as substrate in presence of NADPH/NADH after 10 mins by fluorescence assay	CHEMBL4182166
Inhibition of Staphylococcus aureus FabI assessed as inhibition of bacterial growth by measuring the NADH consumption rate at 20 mins	CHEMBL5231305
Inhibition of Staphylococcus aureus FabI in the presence of NADH using crotonyl CoA as substrate by measuring NADH consumption rate	CHEMBL5231308
Inhibition of Staphylococcus aureus ATCC 29213 FabI in the presence of NADH using crotonyl CoA as substrate by measuring NADH consumption rate	CHEMBL5231344
Inhibition of Staphylococcus aureus FabI assessed as inhibition at 10 uM	CHEMBL5231370

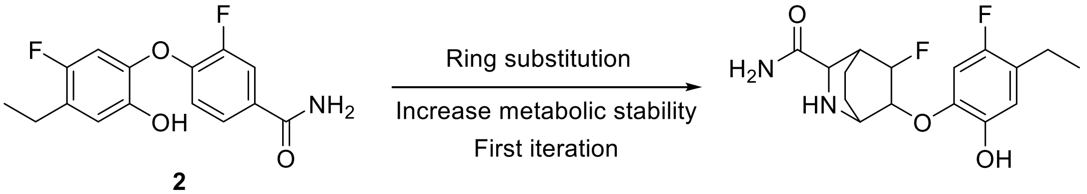

2

SMARTS: c;x2:5]1[c;x2:6][c;x2;D3:1][c;x2:2][c;x2D2:3][c;x2:4]1>>[#6]-1-[#6]-[#6:4]-2-[#6:5]-[#6:6]-[#6:1]-1-[#6:2]-[#8:3]-2|rb:0:2,1:2,2:2,3:2,4:2,5:2,s:4:2|

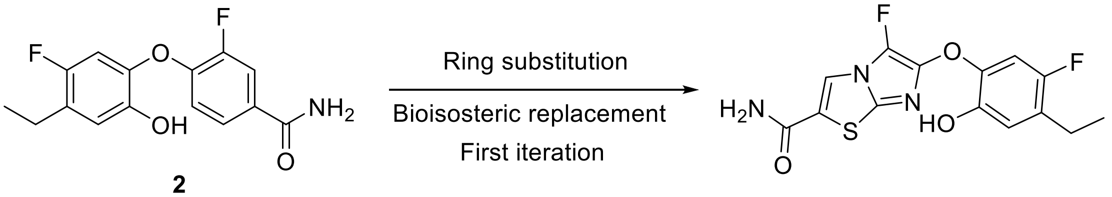

2

SMARTS: [c;x2:1]1[c;x2:6][c;x2:5][c;x2:4][c;x2D2:3][c;x2D2:2]1>>[#16:3]-1-[#6:4]=[#6:5]-[#7]-2-[#6:6]=[#6:1]-[#7:2]=[#6]-1-2|t:12,rb:0:2,2:2,3:2,4:2,5:2,s:4:,5:2|

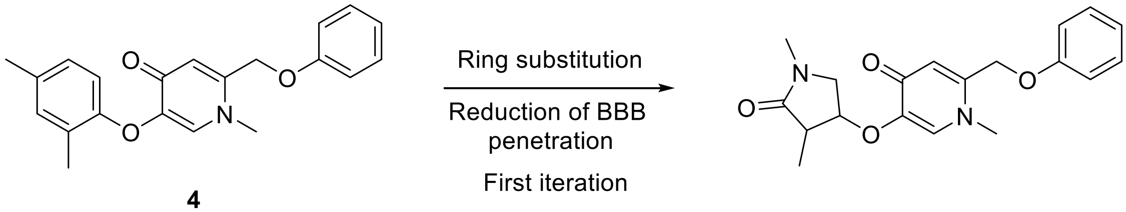

4

SMARTS: [*:6]-[c;x2D3:1]1[c;x2:5][c;x2D2][c;x2:4][c;x2:3][c;x2;D2:2]1>>[*:6]-[#7:1]-1-[#6:5]-[#6:4]-[#6:3]-[#6:2]-1=O|rb:1:2,2:2,3:2,4:2,5:2,6:2,s:1:3|

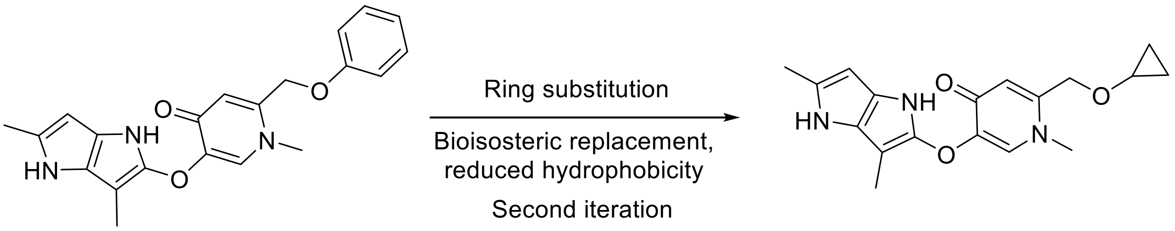

SMARTS: [c;D2,D3;x2:3]1[c;x2D2][c;x2D2][c;x2D3:1][c;x2D2][c;D2,D3;x2:2]1>>[#6:3]-1-[#6:1]-[#6:2]-1

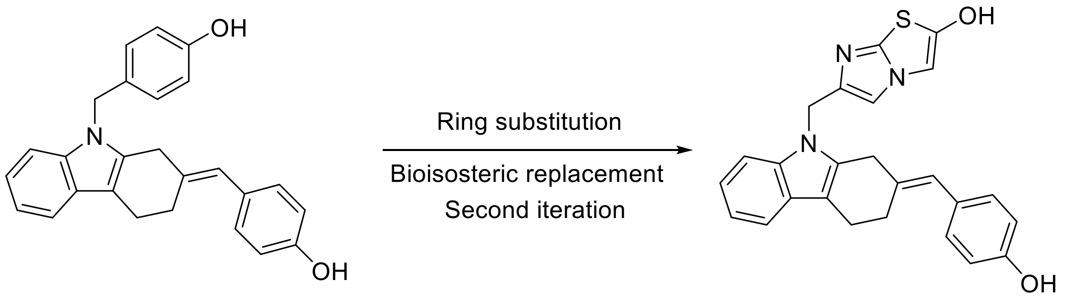

SMARTS: [c;x2:1]1[c;x2:6][c;x2:5][c;x2:4][c;x2D2:3][c;x2D2:2]1>>[#16:3]-1-[#6:4]=[#6:5]-[#7]-2-[#6:6]=[#6:1]-[#7:2]=[#6]-1-2|t:12,rb:0:2,2:2,3:2,4:2,5:2,s:4:2

Figure S3. Examples of transformation rules applied to structures **2**-**4**. The parent structure **2** was modified using transformation rules to improve metabolic stability (row 1) or through bioisosteric replacement (row 2), as reported in drug design. Structure **4** was altered to increase polarity (row 3). The final examples show structures generated from parent structures **4** and **3**, where, in a second iteration, the transformations were aimed at reducing hydrophobicity and at performing a bioisosteric replacement, respectively.

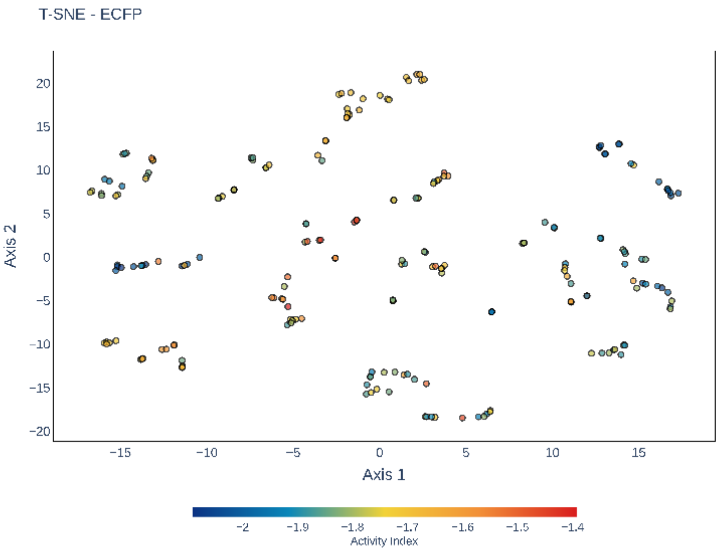

Figure S4. Visualization of the chemical space of STADS using t-SNE based on ECFP4 (r = 2, 2048-bits) fingerprint calculated with RDkit. Dots were colored by pIC_{50}.

Table S2. Statistics of significant pharmaceutical properties and constitutional descriptors in STADS.

Descriptor	$\bar{x}$	Q_2	Q_3
MW	376.96	363.19	402.27
HBA	5.21	5.0	6.0
HBD	0.88	0	1.0
Log (P)	3.47	3.52	4.87
TPSA	65.99	62.63	75.52
#RoB	4.34	4.0	5.0
MR	103.36	98.28	113.29
MC	0.77	0.76	0.83
SI	0.61	0.62	0.66
FractionCSP3	0.23	0.22	0.31
HetAtoms	6.02	6.0	6.0
NumSaturatedRings	0.44	0	1.0
numAliphaticHeterocycles	0.88	1.0	2.0
NumAromaticRings	2.77	3.0	3.0
NumRings	3.71	3.0	4.0
NumAromaticHeterocycles	1.18	1.0	2.0
NumAmideBonds	1.05	1.0	2.0

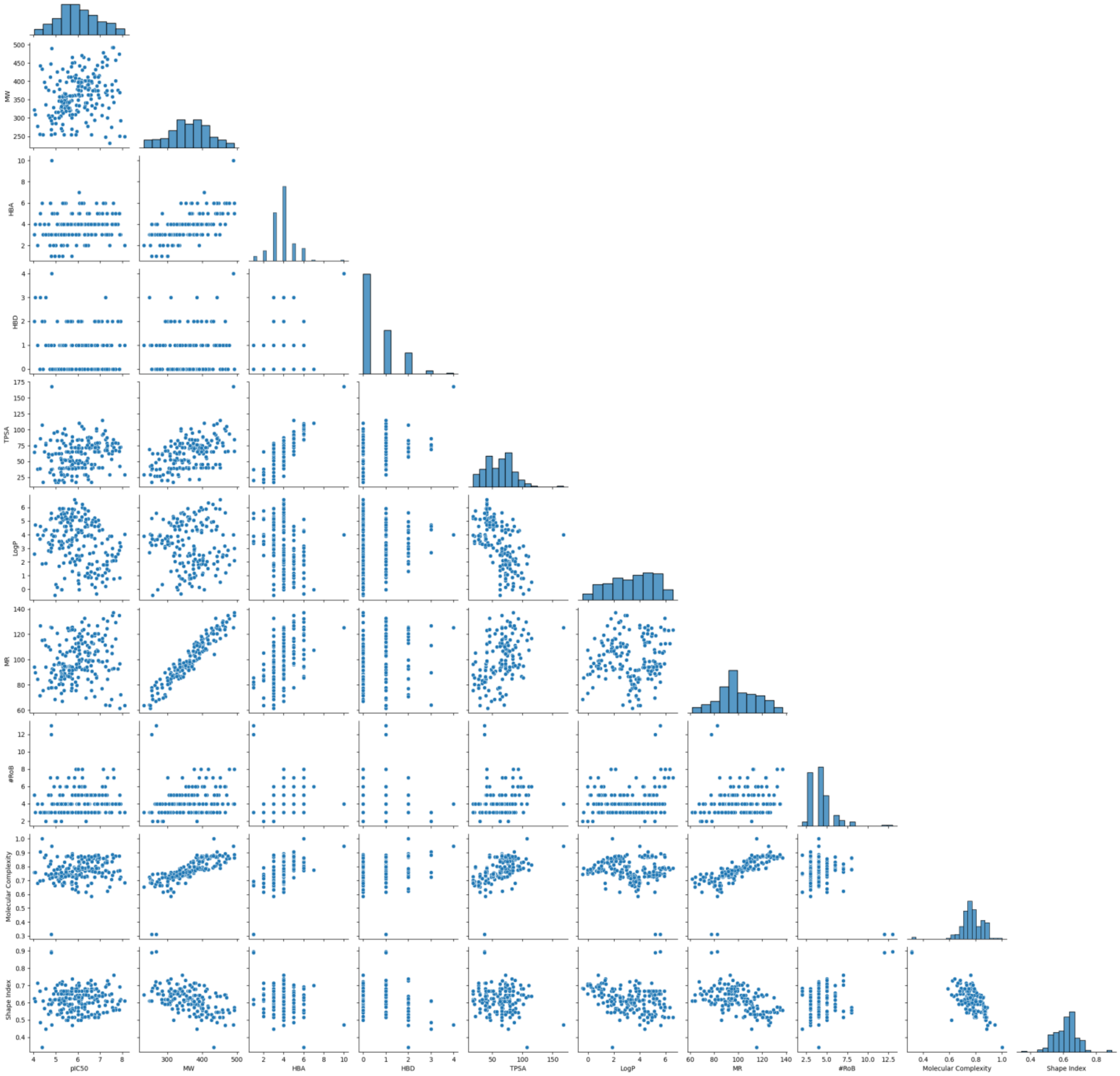

Figure S5. Distribution and dispersion plots of descriptors for the *S. aureus* dataset. Abbreviations: Molecular Weight (MW; g/mol), Hydrogen Bond Donors (HBD), Hydrogen Bond Acceptors (HBA), Topological Polar Surface Area (TPSA; $Å^2$), Octanol-Water Partition Coefficient (Log P), Molar Refractivity (MR; $Å^3/mol$), Number of Rotatable Bonds (nRoB), Molecular Complexity (MC), and Shape Index (SI).

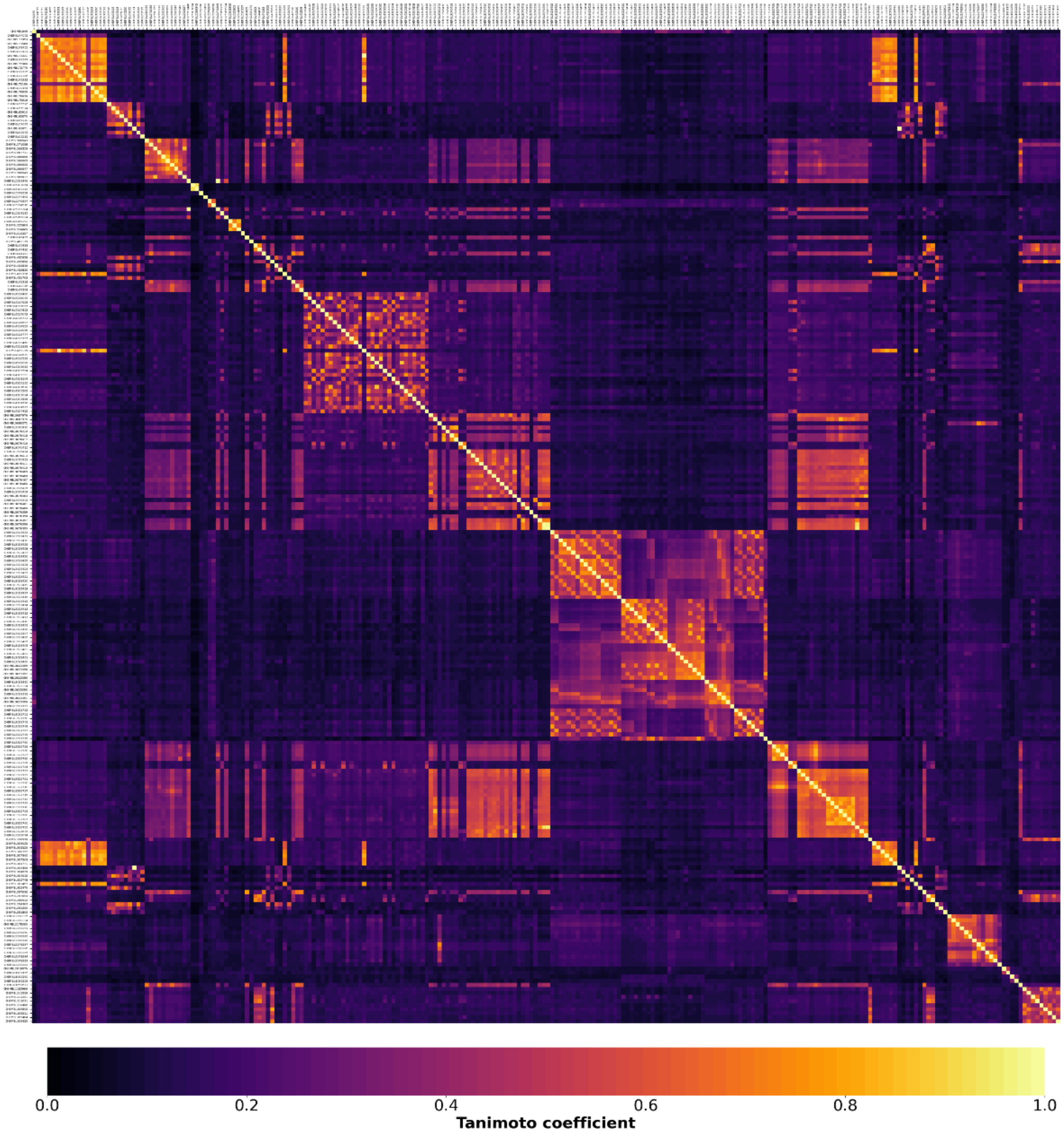

Figure S6. Analysis of structure similarity in the dataset of inhibitors of *S. aureus* FabI given by Tanimoto coefficient using ECFP4 (r = 2, 2048-bits).

Table S3. Pair-wise comparison of chemical structures in STADS with the highest similarity (Tanimoto coefficient) and SALI values greater than five, using ECFP4 fingerprint and ΔpIC_{50}, as utilized in the SAS MAP with a threshold of 75% (quantiles Q_3 of ECFP4 and ΔpIC_{50}).

Compound A	pIC_{50} A	Smiles A	Compound B	pIC_{50} B	Smiles B	Activity difference	Similarity	SALI value
CHEMBL72164	5.0605	O=C(O)c1ccc(Cn2c3c(c4ccccc42)CCN(C(=O)c2ccc(O)cc2)C3)cc1	CHEMBL72232	6.9586	O=C(c1ccc(O)cc1)N1CCc2c(n(Cc3ccc(O)cc3)c3ccccc23)C1	1.8981	0.9184	23.261
CHEMBL433326	4.4895	O=C(c1ccc(O)cc1)N1CCc2c(c3ccccc3n2Cc2ccc(O)cc2)C1	CHEMBL72232	6.9586	O=C(c1ccc(O)cc1)N1CCc2c(n(Cc3ccc(O)cc3)c3ccccc23)C1	2.4692	0.8367	15.1206
CHEMBL3676397	5.2757	O=C1CCc2cc(=CCC(=O)N3CCC(CCO)CC3)cnc2=N1	CHEMBL3676401	7.3872	CCCC1CCN(C(=O)CC=c2cnc3c(c2)CCC(=O)N=3)CC1	2.1115	0.8462	13.7289
CHEMBL2178283	4.6778	CCc1cc(OC)c(Oc2ccc(C(N)=O)cc2F)cc1F	CHEMBL2178284	7.9208	CCc1cc(O)c(Oc2ccc(C(N)=O)cc2F)cc1F	3.243	0.75	12.972
CHEMBL72164	5.0605	O=C(O)c1ccc(Cn2c3c(c4ccccc42)CCN(C(=O)c2ccc(O)cc2)C3)cc1	CHEMBL307960	6.7447	O=C(c1ccc(Cl)cc1)N1CCc2c(n(Cc3ccc(O)cc3)c3ccccc23)C1	1.6842	0.8491	11.161
CHEMBL3680372	5.301	CCc1cc(OC(=O)OC)c(Oc2ccc(C(=O)N3CCNC(=O)C3)cc2F)cc1F	CHEMBL2178287	7.7959	CCc1cc(O)c(Oc2ccc(C(=O)N3CCNC(=O)C3)cc2F)cc1F	2.4949	0.7581	10.3138
CHEMBL433326	4.4895	O=C(c1ccc(O)cc1)N1CCc2c(c3ccccc3n2Cc2ccc(O)cc2)C1	CHEMBL307960	6.7447	O=C(c1ccc(Cl)cc1)N1CCc2c(n(Cc3ccc(O)cc3)c3ccccc23)C1	2.2553	0.7736	9.9616
CHEMBL72164	5.0605	O=C(O)c1ccc(Cn2c3c(c4ccccc42)CCN(C(=O)c2ccc(O)cc2)C3)cc1	CHEMBL70618	6.6198	Cc1ccc(C(=O)N2CCc3c(n(Cc4ccc(O)cc4)c4ccccc34)C2)cc1	1.5593	0.8333	9.3539
CHEMBL72164	5.0605	O=C(O)c1ccc(Cn2c3c(c4ccccc42)CCN(C(=O)c2ccc(O)cc2)C3)cc1	CHEMBL72770	6.6021	O=C(c1ccc(O)cc1)N1CCc2c(n(Cc3ccc(F)cc3)c3ccccc23)C1	1.5416	0.8333	9.2478
CHEMBL433326	4.4895	O=C(c1ccc(O)cc1)N1CCc2c(c3ccccc3n2Cc2ccc(O)cc2)C1	CHEMBL70618	6.6198	Cc1ccc(C(=O)N2CCc3c(n(Cc4ccc(O)cc4)c4ccccc34)C2)cc1	2.1303	0.7593	8.8504
CHEMBL3322705	4.9586	O=C1CCc2cc(=CCC(=O)N3CCC3)cnc2=N1	CHEMBL3676401	7.3872	CCCC1CCN(C(=O)CC=c2cnc3c(c2)CCC(=O)N=3)CC1	2.4286	0.7255	8.8474
CHEMBL433326	4.4895	O=C(c1ccc(O)cc1)N1CCc2c(c3ccccc3n2Cc2ccc(O)cc2)C1	CHEMBL72770	6.6021	O=C(c1ccc(O)cc1)N1CCc2c(n(Cc3ccc(F)cc3)c3ccccc23)C1	2.1126	0.7593	8.7769
CHEMBL73421	4.301	Cc1cc(O)c(C)c(O)c1C(=O)N1CCc2c(n(Cc3ccc(O)cc3)c3ccccc23)C1	CHEMBL73953	6.7447	Cc1cc(C(=O)N2CCc3c(n(Cc4ccc(O)cc4)c4ccccc34)C2)ccc1O	2.4437	0.7188	8.6903
CHEMBL307563	5.4685	O=C(c1cccc(O)c1)N1CCc2c(n(Cc3ccc(O)cc3)c3ccccc23)C1	CHEMBL72232	6.9586	O=C(c1ccc(O)cc1)N1CCc2c(n(Cc3ccc(O)cc3)c3ccccc23)C1	1.4901	0.8269	8.6083
CHEMBL73421	4.301	Cc1cc(O)c(C)c(O)c1C(=O)N1CCc2c(n(Cc3ccc(O)cc3)c3ccccc23)C1	CHEMBL72900	6.9208	Cc1ccc(C(=O)N2CCc3c(n(Cc4ccc(O)cc4)c4ccccc34)C2)c(O)c1	2.6198	0.6923	8.5141
CHEMBL433326	4.4895	O=C(c1ccc(O)cc1)N1CCc2c(c3ccccc3n2Cc2ccc(O)cc2)C1	CHEMBL70636	6.3098	O=C(c1ccccc1)N1CCc2c(n(Cc3ccc(O)cc3)c3ccccc23)C1	1.8203	0.7843	8.439
CHEMBL3676414	5.0655	CCCCCS(=O)(=O)C1CN(C(=O)CC=c2cnc3c(c2)CCC(=O)N=3)C1	CHEMBL3676408	7.6021	CCCCCOC1CN(C(=O)CC=c2cnc3c(c2)CCC(=O)N=3)C1	2.5366	0.6984	8.4105
CHEMBL73421	4.301	Cc1cc(O)c(C)c(O)c1C(=O)N1CCc2c(n(Cc3ccc(O)cc3)c3ccccc23)C1	CHEMBL72232	6.9586	O=C(c1ccc(O)cc1)N1CCc2c(n(Cc3ccc(O)cc3)c3ccccc23)C1	2.6576	0.6833	8.3915
CHEMBL3676396	5.0044	O=C1CCc2cc(=CCC(=O)N3CCCCC3)cnc2=N1	CHEMBL3676401	7.3872	CCCC1CCN(C(=O)CC=c2cnc3c(c2)CCC(=O)N=3)CC1	2.3829	0.7115	8.2596
CHEMBL3676395	5.2596	O=C1CCc2cc(=CCC(=O)N3CCCC3)cnc2=N1	CHEMBL3676401	7.3872	CCCC1CCN(C(=O)CC=c2cnc3c(c2)CCC(=O)N=3)CC1	2.1276	0.7255	7.7508
CHEMBL433326	4.4895	O=C(c1ccc(O)cc1)N1CCc2c(c3ccccc3n2Cc2ccc(O)cc2)C1	CHEMBL73580	6.7959	O=C(c1ccc(O)c(Cl)c1)N1CCc2c(n(Cc3ccc(O)cc3)c3ccccc23)C1	2.3064	0.6897	7.4328
CHEMBL42318	5.6383	CN(Cc1cc2ccccc2n1C)C(=O)CC=c1cnc2c(c1)CCCN=2	CHEMBL44183	7.301	CN(Cc1cc2ccccc2n1C)C(=O)CC=c1cnc2c(c1)CCC(=O)N=2	1.6628	0.7742	7.364
CHEMBL73421	4.301	Cc1cc(O)c(C)c(O)c1C(=O)N1CCc2c(n(Cc3ccc(O)cc3)c3ccccc23)C1	CHEMBL70618	6.6198	Cc1ccc(C(=O)N2CCc3c(n(Cc4ccc(O)cc4)c4ccccc34)C2)cc1	2.3188	0.6825	7.3033
CHEMBL72164	5.0605	O=C(O)c1ccc(Cn2c3c(c4ccccc42)CCN(C(=O)c2ccc(O)cc2)C3)cc1	CHEMBL73580	6.7959	O=C(c1ccc(O)c(Cl)c1)N1CCc2c(n(Cc3ccc(O)cc3)c3ccccc23)C1	1.7354	0.7586	7.1889
CHEMBL73421	4.301	Cc1cc(O)c(C)c(O)c1C(=O)N1CCc2c(n(Cc3ccc(O)cc3)c3ccccc23)C1	CHEMBL309025	6.7959	O=C(c1ccc(Cl)cc1O)N1CCc2c(n(Cc3ccc(O)cc3)c3ccccc23)C1	2.4949	0.6515	7.159
CHEMBL73421	4.301	Cc1cc(O)c(C)c(O)c1C(=O)N1CCc2c(n(Cc3ccc(O)cc3)c3ccccc23)C1	CHEMBL73580	6.7959	O=C(c1ccc(O)c(Cl)c1)N1CCc2c(n(Cc3ccc(O)cc3)c3ccccc23)C1	2.4949	0.6515	7.159
CHEMBL433326	4.4895	O=C(c1ccc(O)cc1)N1CCc2c(c3ccccc3n2Cc2ccc(O)cc2)C1	CHEMBL73953	6.7447	Cc1cc(C(=O)N2CCc3c(n(Cc4ccc(O)cc4)c4ccccc34)C2)ccc1O	2.2553	0.678	7.004
CHEMBL433326	4.4895	O=C(c1ccc(O)cc1)N1CCc2c(c3ccccc3n2Cc2ccc(O)cc2)C1	CHEMBL421119	6.1739	Nc1ccc(Cn2c3c(c4ccccc42)CCN(C(=O)c2ccc(O)cc2)C3)cc1	1.6845	0.7593	6.9983
CHEMBL433326	4.4895	O=C(c1ccc(O)cc1)N1CCc2c(c3ccccc3n2Cc2ccc(O)cc2)C1	CHEMBL302497	6.4318	COc1ccc(C(=O)N2CCc3c(n(Cc4ccc(O)cc4)c4ccccc34)C2)cc1	1.9423	0.7193	6.9195
CHEMBL73421	4.301	Cc1cc(O)c(C)c(O)c1C(=O)N1CCc2c(n(Cc3ccc(O)cc3)c3ccccc23)C1	CHEMBL307960	6.7447	O=C(c1ccc(Cl)cc1)N1CCc2c(n(Cc3ccc(O)cc3)c3ccccc23)C1	2.4437	0.6406	6.7994

CHEMBL433326	4.4895	O=C(c1ccc(O)cc1)N1CCc2c(c3ccccc3n2Cc2ccc(O)cc2)C1	CHEMBL308352	6.4815	CS(=O)(=O)c1ccc(Cn2c3c(c4ccccc42)CCN(C(=O)c2ccc(O)cc2)C3)cc1	1.992	0.7069	6.7963
CHEMBL3322705	4.9586	O=C1CCc2cc(=CCC(=O)N3CCC3)cnc2=N1	CHEMBL3676405	7.8861	O=C1CCc2cc(=CCC(=O)N3CC(OCc4cccs4)C3)cnc2=N1	2.9274	0.5645	6.7219
CHEMBL3322705	4.9586	O=C1CCc2cc(=CCC(=O)N3CCC3)cnc2=N1	CHEMBL3676408	7.6021	CCCCCOC1CN(C(=O)CC=c2cnc3c(c2)CCC(=O)N=3)C1	2.6435	0.6034	6.6654
CHEMBL72164	5.0605	O=C(O)c1ccc(Cn2c3c(c4ccccc42)CCN(C(=O)c2ccc(O)cc2)C3)cc1	CHEMBL73953	6.7447	Cc1cc(C(=O)N2CCc3c(n(Cc4ccc(O)cc4)c4ccccc34)C2)ccc1O	1.6842	0.7458	6.6255
CHEMBL3676396	5.0044	O=C1CCc2cc(=CCC(=O)N3CCCCC3)cnc2=N1	CHEMBL3676405	7.8861	O=C1CCc2cc(=CCC(=O)N3CC(OCc4cccs4)C3)cnc2=N1	2.8817	0.5556	6.4845
CHEMBL3680372	5.301	CCc1cc(OC(=O)OC)c(Oc2ccc(C(=O)N3CCNC(=O)C3)cc2F)cc1F	CHEMBL2178286	7.5528	CCc1ccc(Oc2ccc(C(=O)N3CCNC(=O)C3)cc2F)c(O)c1	2.2518	0.6515	6.4614
CHEMBL3676396	5.0044	O=C1CCc2cc(=CCC(=O)N3CCCCC3)cnc2=N1	CHEMBL3676408	7.6021	CCCCCOC1CN(C(=O)CC=c2cnc3c(c2)CCC(=O)N=3)C1	2.5977	0.5932	6.3857
CHEMBL433326	4.4895	O=C(c1ccc(O)cc1)N1CCc2c(c3ccccc3n2Cc2ccc(O)cc2)C1	CHEMBL73331	6	Nc1ccc(C(=O)N2CCc3c(n(Cc4ccc(O)cc4)c4ccccc34)C2)cc1	1.5105	0.7593	6.2754
CHEMBL73421	4.301	Cc1cc(O)c(C)c(O)c1C(=O)N1CCc2c(n(Cc3ccc(O)cc3)c3ccccc23)C1	CHEMBL72770	6.6021	O=C(c1ccc(O)cc1)N1CCc2c(n(Cc3ccc(F)cc3)c3ccccc23)C1	2.301	0.6308	6.2324
CHEMBL2178283	4.6778	CCc1cc(OC)c(Oc2ccc(C(N)=O)cc2F)cc1F	CHEMBL2178304	8.0969	CCc1cc(O)c(Oc2ccccc2F)cc1F	3.4191	0.449	6.2053
CHEMBL433326	4.4895	O=C(c1ccc(O)cc1)N1CCc2c(c3ccccc3n2Cc2ccc(O)cc2)C1	CHEMBL72947	6.2924	CCCCc1ccc(C(=O)N2CCc3c(n(Cc4ccc(O)cc4)c4ccccc34)C2)cc1	1.803	0.7069	6.1515
CHEMBL73421	4.301	Cc1cc(O)c(C)c(O)c1C(=O)N1CCc2c(n(Cc3ccc(O)cc3)c3ccccc23)C1	CHEMBL70636	6.3098	O=C(c1ccccc1)N1CCc2c(n(Cc3ccc(O)cc3)c3ccccc23)C1	2.0088	0.6721	6.1263
CHEMBL3676414	5.0655	CCCCCS(=O)(=O)C1CN(C(=O)CC=c2cnc3c(c2)CCC(=O)N=3)C1	CHEMBL3676401	7.3872	CCCC1CCN(C(=O)CC=c2cnc3c(c2)CCC(=O)N=3)CC1	2.3217	0.619	6.0937
CHEMBL431763	4.4377	Cc1ccccc1Cn1cnc(-c2ccsc2)c1	CHEMBL64645	6.6021	Cc1ccc(Cn2cnc(-c3ccsc3)c2)cc1	2.1644	0.6444	6.0866
CHEMBL3676395	5.2596	O=C1CCc2cc(=CCC(=O)N3CCCC3)cnc2=N1	CHEMBL3676405	7.8861	O=C1CCc2cc(=CCC(=O)N3CC(OCc4cccs4)C3)cnc2=N1	2.6264	0.5645	6.0308
CHEMBL433326	4.4895	O=C(c1ccc(O)cc1)N1CCc2c(c3ccccc3n2Cc2ccc(O)cc2)C1	CHEMBL72900	6.9208	Cc1ccc(C(=O)N2CCc3c(n(Cc4ccc(O)cc4)c4ccccc34)C2)c(O)c1	2.4314	0.5968	6.0303
CHEMBL3322705	4.9586	O=C1CCc2cc(=CCC(=O)N3CCC3)cnc2=N1	CHEMBL3322675	7.301	O=C1CCc2cc(=CCC(=O)N3CC(OCc4ccccc4)C3)cnc2=N1	2.3424	0.6034	5.9062
CHEMBL3676395	5.2596	O=C1CCc2cc(=CCC(=O)N3CCCC3)cnc2=N1	CHEMBL3676408	7.6021	CCCCCOC1CN(C(=O)CC=c2cnc3c(c2)CCC(=O)N=3)C1	2.3424	0.6034	5.9062
CHEMBL3676414	5.0655	CCCCCS(=O)(=O)C1CN(C(=O)CC=c2cnc3c(c2)CCC(=O)N=3)C1	CHEMBL3676405	7.8861	O=C1CCc2cc(=CCC(=O)N3CC(OCc4cccs4)C3)cnc2=N1	2.8206	0.5205	5.8824
CHEMBL433326	4.4895	O=C(c1ccc(O)cc1)N1CCc2c(c3ccccc3n2Cc2ccc(O)cc2)C1	CHEMBL309025	6.7959	O=C(c1ccc(Cl)cc1O)N1CCc2c(n(Cc3ccc(O)cc3)c3ccccc23)C1	2.3064	0.6066	5.8627
CHEMBL109484	4.0283	CN(Cc1cc2ccccc2n1C)C(=O)CCc1ccc(=N)[nH]c1	CHEMBL44183	7.301	CN(Cc1cc2ccccc2n1C)C(=O)CC=c1cnc2c(c1)CCC(=O)N=2	3.2728	0.4306	5.7478
CHEMBL3676397	5.2757	O=C1CCc2cc(=CCC(=O)N3CCC(CCO)CC3)cnc2=N1	CHEMBL3676405	7.8861	O=C1CCc2cc(=CCC(=O)N3CC(OCc4cccs4)C3)cnc2=N1	2.6103	0.5441	5.7256
CHEMBL73421	4.301	Cc1cc(O)c(C)c(O)c1C(=O)N1CCc2c(n(Cc3ccc(O)cc3)c3ccccc23)C1	CHEMBL302497	6.4318	COc1ccc(C(=O)N2CCc3c(n(Cc4ccc(O)cc4)c4ccccc34)C2)cc1	2.1308	0.6269	5.7111
CHEMBL73421	4.301	Cc1cc(O)c(C)c(O)c1C(=O)N1CCc2c(n(Cc3ccc(O)cc3)c3ccccc23)C1	CHEMBL72286	6.1427	O=C(c1ccccc1O)N1CCc2c(n(Cc3ccc(O)cc3)c3ccccc23)C1	1.8416	0.6774	5.7086
CHEMBL73421	4.301	Cc1cc(O)c(C)c(O)c1C(=O)N1CCc2c(n(Cc3ccc(O)cc3)c3ccccc23)C1	CHEMBL308352	6.4815	CS(=O)(=O)c1ccc(Cn2c3c(c4ccccc42)CCN(C(=O)c2ccc(O)cc2)C3)cc1	2.1805	0.6176	5.7021
CHEMBL294363	5.1158	c1ccc(-c2ccc(Cn3cnc(-c4ccsc4)c3)cc2)cc1	CHEMBL64645	6.6021	Cc1ccc(Cn2cnc(-c3ccsc3)c2)cc1	1.4863	0.7381	5.6751
CHEMBL3623430	5.5702	Cc1ccc(Oc2cn(Cc3ccccc3)c(COc3ccccc3)cc2=O)c(C)c1	CHEMBL3623427	7.0969	Cc1ccc(Oc2cn(C)c(COc3ccccc3)cc2=O)c(C)c1	1.5267	0.7308	5.6712
CHEMBL3623428	5.4989	Cc1ccc(Oc2cn(C3CC3)c(COc3ccccc3)cc2=O)c(C)c1	CHEMBL3623427	7.0969	Cc1ccc(Oc2cn(C)c(COc3ccccc3)cc2=O)c(C)c1	1.598	0.717	5.6466
CHEMBL3676396	5.0044	O=C1CCc2cc(=CCC(=O)N3CCCCC3)cnc2=N1	CHEMBL3322675	7.301	O=C1CCc2cc(=CCC(=O)N3CC(OCc4ccccc4)C3)cnc2=N1	2.2967	0.5932	5.6458

CHEMBL2178283	4.6778	CCc1cc(OC)c(Oc2ccc(C(N)=O)cc2F)cc1F	CHEMBL2178287	7.7959	CCc1cc(O)c(Oc2ccc(C(=O)N3CCNC(=O)C3)cc2F)cc1F	3.1181	0.4444	5.6121
CHEMBL3322674	6	O=C1CCc2cc(=CCC(=O)N3CC(Oc4ccccc4)C3)cnc2=N1	CHEMBL3676405	7.8861	O=C1CCc2cc(=CCC(=O)N3CC(OCc4cccs4)C3)cnc2=N1	1.8861	0.6615	5.5719
CHEMBL72164	5.0605	O=C(O)c1ccc(Cn2c3c(c4ccccc42)CCN(C(=O)c2ccc(O)cc2)C3)cc1	CHEMBL72900	6.9208	Cc1ccc(C(=O)N2CCc3c(n(Cc4ccc(O)cc4)c4ccccc34)C2)c(O)c1	1.8603	0.6613	5.4925
CHEMBL3676400	5.1487	O=C1CCc2cc(=CCC(=O)N3CCCC3c3ccccc3)cnc2=N1	CHEMBL3676405	7.8861	O=C1CCc2cc(=CCC(=O)N3CC(OCc4cccs4)C3)cnc2=N1	2.7373	0.4932	5.4011
CHEMBL3322705	4.9586	O=C1CCc2cc(=CCC(=O)N3CCC3)cnc2=N1	CHEMBL3676412	7.2441	O=C1CCc2cc(=CCC(=O)N3CC(OCc4nccs4)C3)cnc2=N1	2.2855	0.5738	5.3625
CHEMBL2178283	4.6778	CCc1cc(OC)c(Oc2ccc(C(N)=O)cc2F)cc1F	CHEMBL2178285	7.0862	CCc1cc(O)c(Oc2ccc(C(=O)NC)cc2F)cc1F	2.4084	0.549	5.3401
CHEMBL72164	5.0605	O=C(O)c1ccc(Cn2c3c(c4ccccc42)CCN(C(=O)c2ccc(O)cc2)C3)cc1	CHEMBL309025	6.7959	O=C(c1ccc(Cl)cc1O)N1CCc2c(n(Cc3ccc(O)cc3)c3ccccc23)C1	1.7354	0.6721	5.2925
CHEMBL3676397	5.2757	O=C1CCc2cc(=CCC(=O)N3CCC(CCO)CC3)cnc2=N1	CHEMBL3676408	7.6021	CCCCCOC1CN(C(=O)CC=c2cnc3c(c2)CCC(=O)N=3)C1	2.3263	0.5538	5.2136
CHEMBL73421	4.301	Cc1cc(O)c(C)c(O)c1C(=O)N1CCc2c(n(Cc3ccc(O)cc3)c3ccccc23)C1	CHEMBL72947	6.2924	CCCCc1ccc(C(=O)N2CCc3c(n(Cc4ccc(O)cc4)c4ccccc34)C2)cc1	1.9914	0.6176	5.2076
CHEMBL2178283	4.6778	CCc1cc(OC)c(Oc2ccc(C(N)=O)cc2F)cc1F	CHEMBL2178291	7.8861	CCc1cc(O)c(Oc2cccnc2F)cc1F	3.2083	0.3818	5.1897
CHEMBL2178283	4.6778	CCc1cc(OC)c(Oc2ccc(C(N)=O)cc2F)cc1F	CHEMBL2178305	7.5086	CCc1cc(O)c(Oc2ccc(C#N)cc2F)cc1F	2.8309	0.4528	5.1734
CHEMBL3676395	5.2596	O=C1CCc2cc(=CCC(=O)N3CCCC3)cnc2=N1	CHEMBL3322675	7.301	O=C1CCc2cc(=CCC(=O)N3CC(OCc4ccccc4)C3)cnc2=N1	2.0414	0.6034	5.1473
CHEMBL4217928	4.5467	CC(=O)N=c1ccc(C=CC(=O)N2CCC(c3ccccn3)C2)c[nH]1	CHEMBL3322724	6.7932	CC(=O)N=c1ccc(C=CC(=O)N2CC(c3oc4ccccc4c3C)C2)c[nH]1	2.2465	0.5634	5.1454
CHEMBL3676396	5.0044	O=C1CCc2cc(=CCC(=O)N3CCCCC3)cnc2=N1	CHEMBL3676412	7.2441	O=C1CCc2cc(=CCC(=O)N3CC(OCc4nccs4)C3)cnc2=N1	2.2398	0.5645	5.1431
CHEMBL3676400	5.1487	O=C1CCc2cc(=CCC(=O)N3CCCC3c3ccccc3)cnc2=N1	CHEMBL3676401	7.3872	CCCC1CCN(C(=O)CC=c2cnc3c(c2)CCC(=O)N=3)CC1	2.2385	0.5625	5.1166
CHEMBL73421	4.301	Cc1cc(O)c(C)c(O)c1C(=O)N1CCc2c(n(Cc3ccc(O)cc3)c3ccccc23)C1	CHEMBL421119	6.1739	Nc1ccc(Cn2c3c(c4ccccc42)CCN(C(=O)c2ccc(O)cc2)C3)cc1	1.8729	0.6308	5.0729
CHEMBL3322705	4.9586	O=C1CCc2cc(=CCC(=O)N3CCC3)cnc2=N1	CHEMBL3687976	6.8239	Cc1nc(C2CN(C(=O)CC=c3cnc4c(c3)CCC(=O)N=4)C2)no1	1.8653	0.6316	5.0632
CHEMBL3322705	4.9586	O=C1CCc2cc(=CCC(=O)N3CCC3)cnc2=N1	CHEMBL3676403	6.9208	O=C1CCc2cc(=CCC(=O)N3CCC(Oc4ccccc4)C3)cnc2=N1	1.9622	0.6102	5.0339
CHEMBL3322705	4.9586	O=C1CCc2cc(=CCC(=O)N3CCC3)cnc2=N1	CHEMBL3676407	6.4685	O=C1CCc2cc(=CCC(=O)N3CCC(O)(c4ccccc4)CC3)cnc2=N1	1.5099	0.6981	5.0013

Table S4. Regression metrics for a support vector regressor built from STADS using different fingerprints.

Fingerprint	MAE[a]	RMSE[b]	R^2 $\boldsymbol{test}$	R^2 $\boldsymbol{train}$
ECFP	0.3707	0.4867	0.8912	0.9684
Topological	0.3877	0.4902	0.8657	0.9407
FCFP	0.3707	0.4867	0.8912	0.9684
Atompair	0.3897	0.4776	0.8864	0.9888
Avalon	0.4577	0.5709	0.8077	0.9558
Layered	0.4491	0.5463	0.8420	0.9566
Pattern	0.4656	0.5943	0.7857	0.9220
MACCS	0.4040	0.5119	0.8464	0.9376

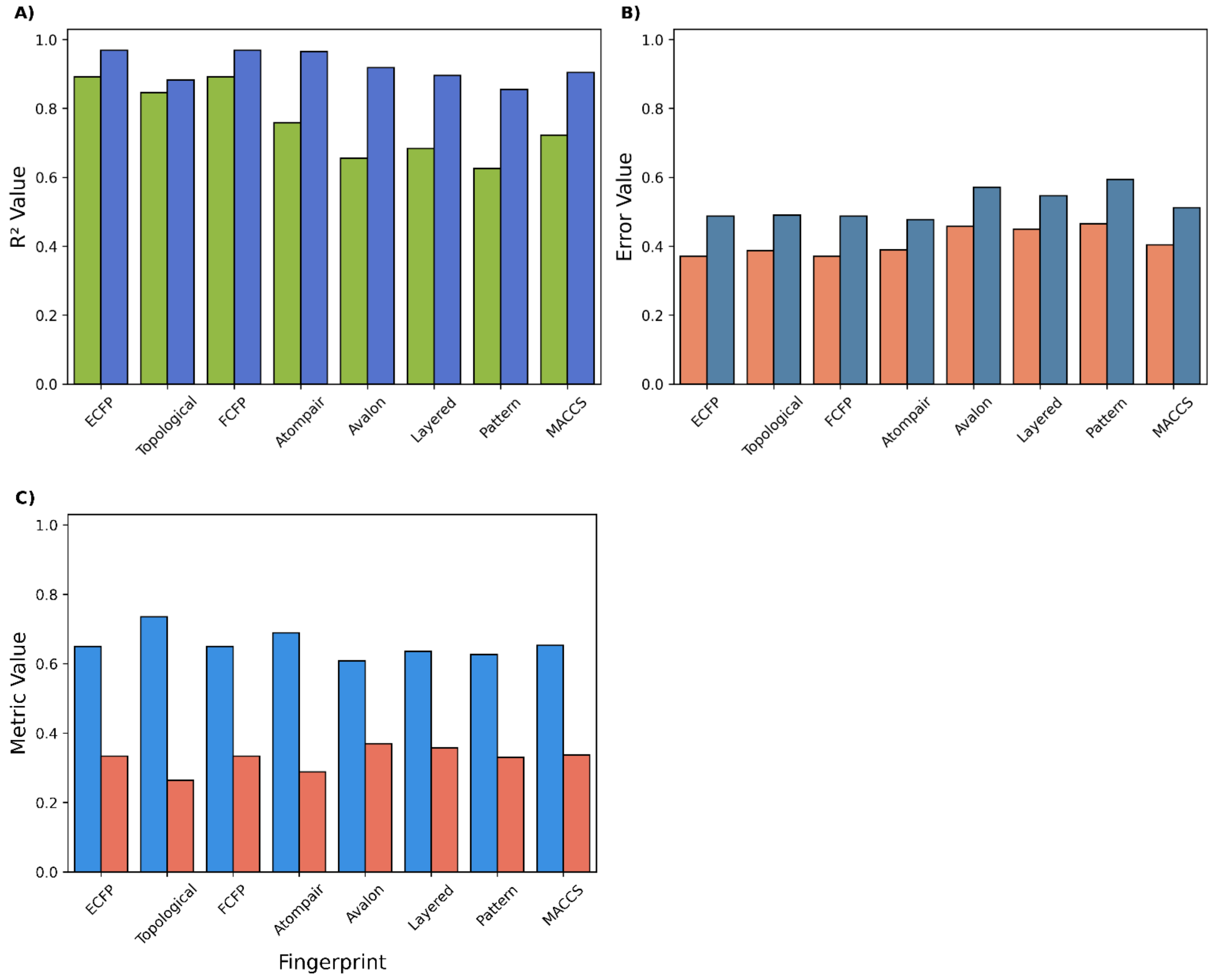

Figure S7. Regression metrics for a support vector regressor built from *S. aureus* dataset, employing different fingerprints. A) The yellow-green bars indicate the Spearman correlation coefficient for the testing set, while the royal-blue bars represent the Spearman correlation coefficient for the training set. B) The coral bars and steel-blue bars highlight the MAE and RMSE metrics, respectively. C) The dodger-blue and coral bars show the results of cross-validation and leave-one-out cross-validation of the MSE, respectively.

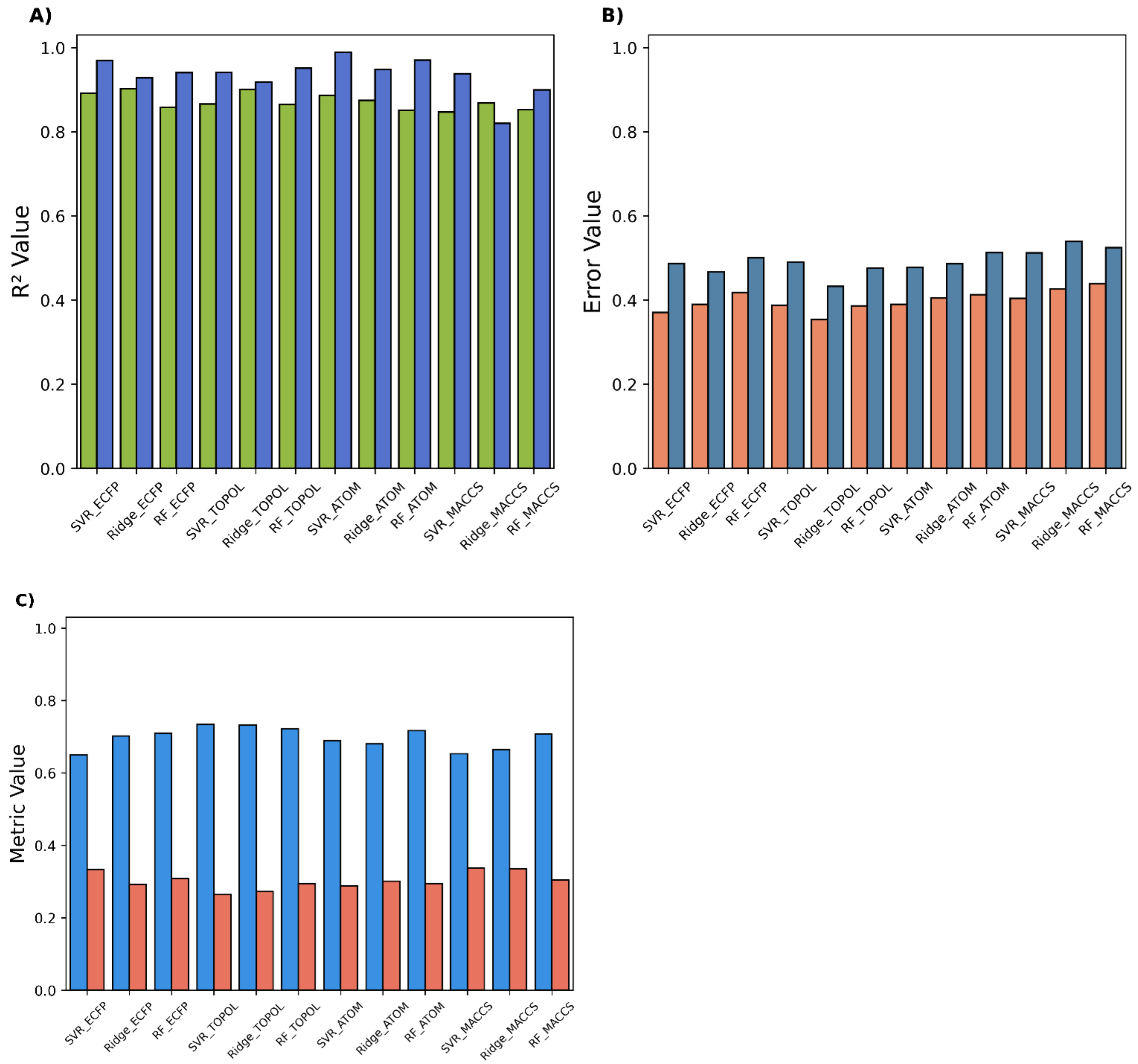

Figure S8. Regression metrics for machine learning models built from *S. aureus* dataset, employing ECFP, Topological torsions, Atompairs and MACCS keys fingerprints. A) The yellow-green bars indicate the Spearman correlation coefficient for the testing set, while the royal-blue bars represent the Spearman correlation coefficient for the training set. B) The coral bars and steel-blue bars highlight the MAE and RMSE metrics, respectively. C) The dodger-blue and coral bars show the results of cross-validation and leave-one-out cross-validation of the MSE, respectively.

Table S5. Hyperparameter optimization in the ML models. R^2 metrics and CV validation were assessed using the Spearman correlation coefficient.

Regressor model	Split FP	Hyperparameters	MAE	RMSE	R^2 test	R^2 train	CV(5)	LOOCV MSE
SVR	No	(C = 3, ε = 0.01, γ = auto, kernel = rbf)	0.3707	0.4867	0.8912	0.9684	0.6501	0.3339
Ridge	No	α = 10, fit_intercept=True, solver='saga'	0.3900	0.4671	0.9019	0.9278	0.7024	0.2926
Bayesian Ridge	Yes	{'alpha_1': 0.1, 'alpha_2': 1e-06, 'lambda_1': 1e-06, 'lambda_2': 0.001}	0.3732	0.4552	0.9040	0.9215	0.6857	0.2950
Random Forest	No	n_estimators': 400, 'min_samples_split': 2, 'min_samples_leaf': 1, 'max_features': 'sqrt', 'max_depth': 10, 'bootstrap': False random_state: 42	0.4180	0.4999	0.8581	0.9401	0.7102	0.3088
Huber	No	alpha=0.01, epsilon=1.1, max_iter=10000, tol=1e-6	0.3967	0.5297	0.8399	0.9764	0.6548	0.3658
K neighbors	No	metric='manhattan', n_jobs=-1, n_neighbors=8	0.4116	0.5208	0.8354	0.9997	0.6757	0.3245

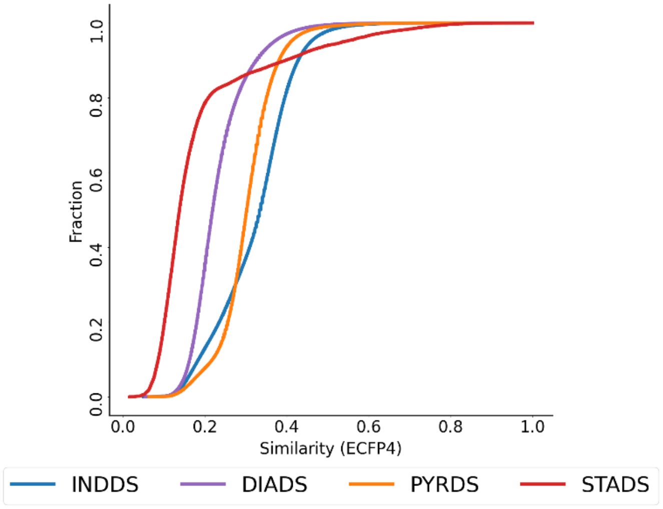

Figure S9. Cumulative distribution function of the pair-wise similarity values of INDDS, DIADS, PYRDS and STADS. Similarity was calculated with RDkit employing ECFP4 (r = 2, 2048-bits) and the Tanimoto coefficient.

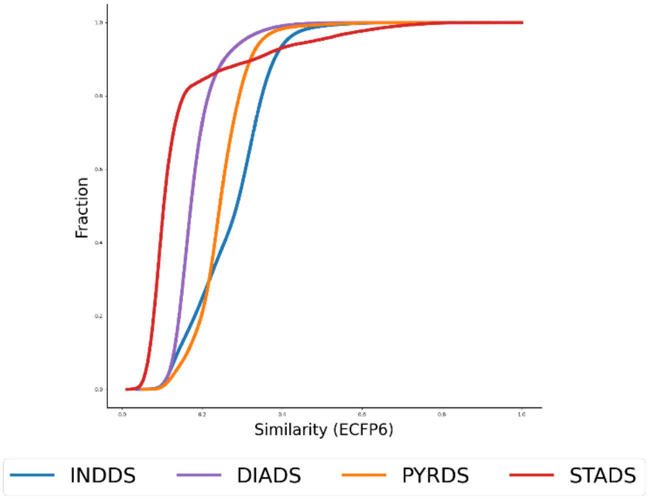

Figure S10. Cumulative distribution function of the pair-wise similarity values of INDDS, DIADS, PYRDS and STADS. Similarity was calculated with RDkit employing ECFP6 (r = 3, 2048-bits) and the Tanimoto coefficient.

Table S6. Summary statistics of the similarity scores calculated using the Tanimoto coefficient for the designed compound libraries and STADS from cumulative distribution functions.

Dataset	Count	Mean	Std	Min	25%	50%	75%	Max
MACCS INDDS	12497500	0.694	0.081	0.272	0.64	0.694	0.75	1
MACCS PYRDS	12497500	0.672	0.083	0.311	0.616	0.671	0.727	1
MACCS DIADS	12497500	0.583	0.102	0.222	0.507	0.575	0.651	1
MACCS STADS	23436	0.473	0.178	0.02	0.338	0.458	0.595	1
ECFP4 INDDS	12497500	0.333	0.092	0.054	0.268	0.346	0.395	1
ECFP4 PYRDS	12497500	0.312	0.069	0.069	0.275	0.311	0.35	0.98
ECFP4 DIADS	12497500	0.243	0.069	0.056	0.197	0.229	0.274	0.889
ECFP4 STADS	23436	0.2	0.141	0.029	0.122	0.155	0.202	1
ECFP6 INDDS	12497500	0.289	0.093	0.037	0.216	0.302	0.354	0.917
ECFP6 PYRDS	12497500	0.26	0.066	0.056	0.221	0.258	0.297	0.942
ECFP6 DIADS	12497500	0.197	0.058	0.045	0.16	0.185	0.218	0.831
ECFP6 STADS	23436	0.166	0.129	0.031	0.101	0.124	0.158	1

Table S7 Generated structures obtained from transformation rules and their presence in the original STADS with ACs. Experimental pIC_{50} and mean $ppIC_{50}$ of all ML models (SVR, RR and RFR) are described along with similarity scores calculated from parent structures **2-4**.

Entry	Structures	Exp. pIC_{50}	Mean $ppIC_{50}$	similarity	dataset
1	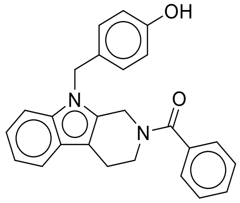	6.3098	6.2161	0.9362	INDDS
2	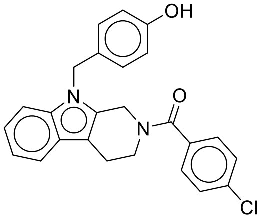	6.7447	6.3705	0.9184	INDDS
3	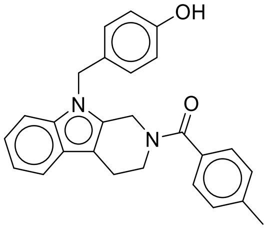	6.6198	6.4509	0.9000	INDDS
4	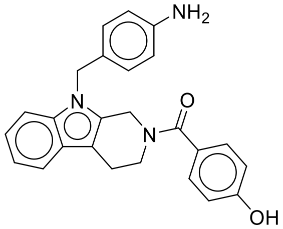	6.1739	6.1330	0.9000	INDDS
5	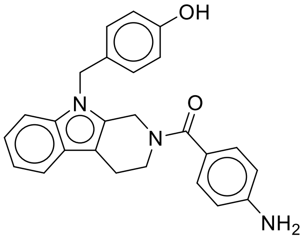	6.0000	6.1330	0.9000	INDDS
6	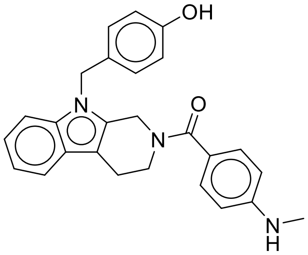	5.6778	5.8739	0.8491	INDDS
7	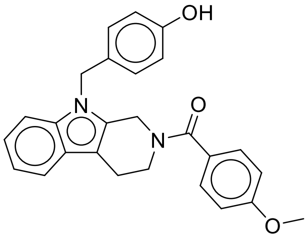	6.4318	6.3103	0.8491	INDDS

8	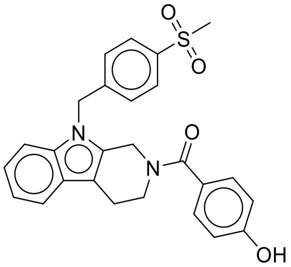	6.4815	6.3536	0.8333	INDDS
9	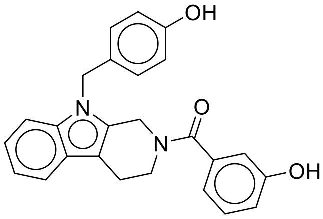	5.4685	6.0975	0.8269	INDDS
10	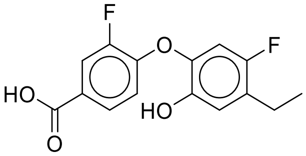	6.7447	7.4045	0.7857	DIADS
11*	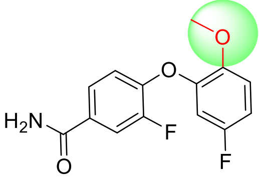 11	4.6778	6.8162	0.7500	DIADS
12	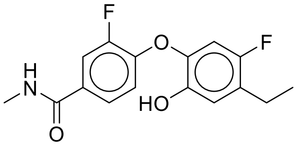	7.0862	7.1300	0.7333	DIADS
13	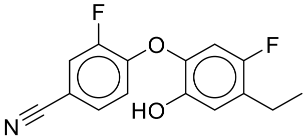	7.5086	7.4805	0.6170	DIADS
14	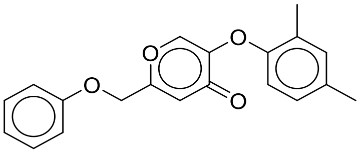	6.4202	6.0264	0.6038	PYRDS
15	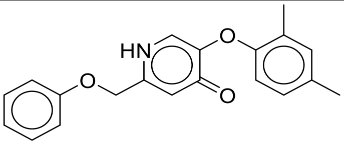	5.9431	5.7781	0.6038	PYRDS
16	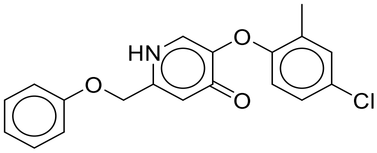	6.3979	5.8556	0.4746	PYRDS

*Activity cliff

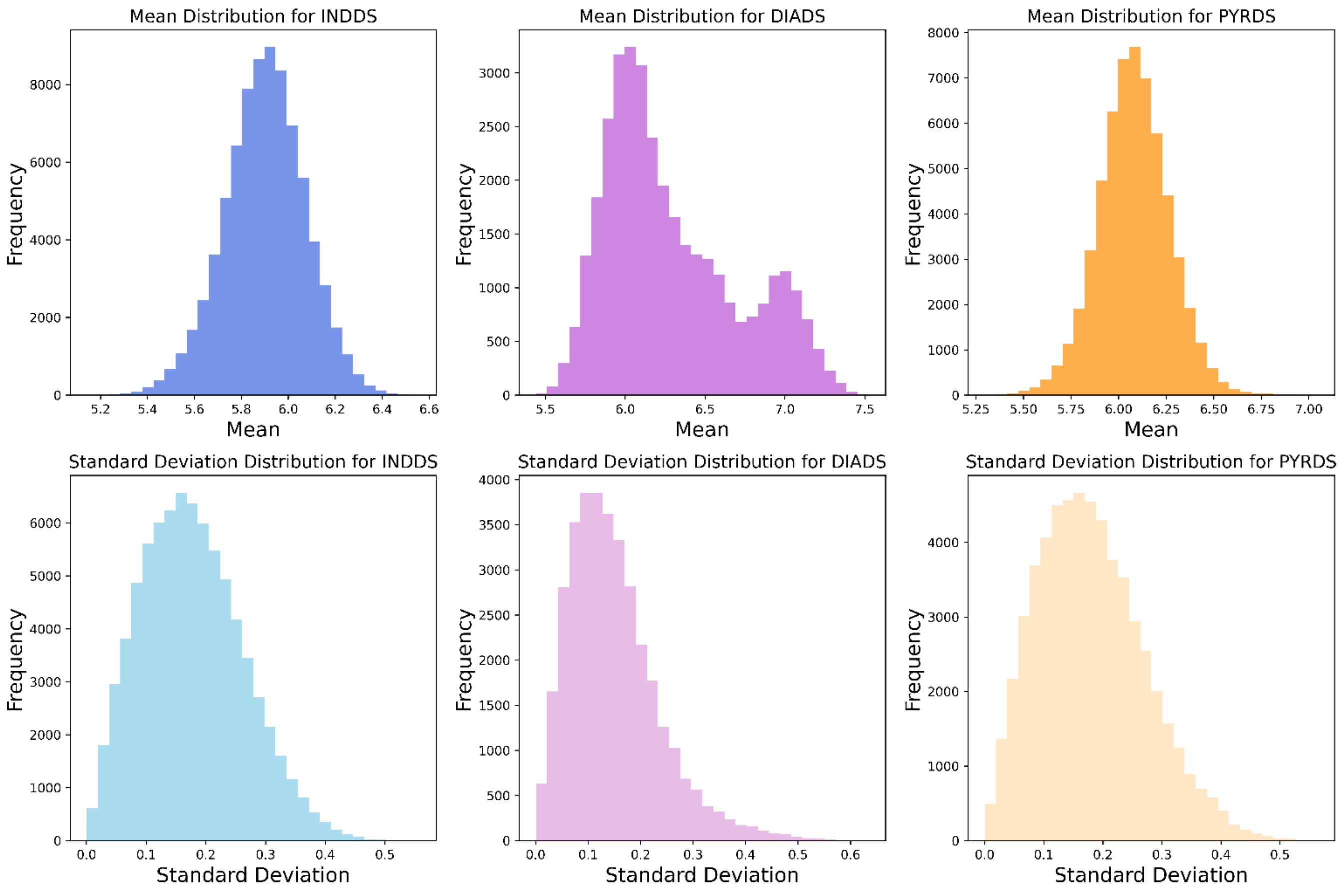

Figure S11. Distributions of mean x̄ values (first row) and standard deviation (second row) of SVR, RR and RFR models utilized by the three designed libraries INDDS, DIADS, and PYRDS. INDDS ($\bar{x} = 5.9004, \sigma = 0.1719$), DIADS ($\bar{x} = 6.2922, \sigma = 0.4181$), PYRDS ($\bar{x} = 6.0808, \sigma = 0.1850$).
